# Nuclear SphK2/S1P signaling is a key regulator of ApoE production and Aβ uptake in astrocytes

**DOI:** 10.1016/j.jlr.2024.100510

**Published:** 2024-01-26

**Authors:** Masato Komai, Yuka Noda, Atsuya Ikeda, Nanaka Kaneshiro, Yuji Kamikubo, Takashi Sakurai, Takashi Uehara, Nobumasa Takasugi

**Affiliations:** 1 Department of Medicinal Pharmacology, Graduate School of Medicine, Dentistry and Pharmaceutical Sciences, Okayama University, Kita, Okayama, Japan; 2 Research Fellow of Japan Society for the Promotion of Science, Chiyoda, Tokyo, Japan; 3 Department of Cellular and Molecular Pharmacology, Juntendo University Graduate School of Medicine, Bunkyo, Tokyo, Japan

**Keywords:** alzheimer’s disease, apolipoproteins, nuclear receptors/RXR, transcription, sphingosine phosphate, astrocytes, amyloid β, sphingosine kinase 2, low-density lipoprotein receptor-related protein 4

## Abstract

The link between changes in astrocyte function and the pathological progression of Alzheimer's disease (AD) has attracted considerable attention. Interestingly, activated astrocytes in AD show abnormalities in their lipid content and metabolism. In particular, the expression of apolipoprotein E (ApoE), a lipid transporter, is decreased. Because ApoE has anti-inflammatory and amyloid β (Aβ)-metabolizing effects, the nuclear receptors, retinoid X receptor (RXR) and LXR, which are involved in ApoE expression, are considered promising therapeutic targets for AD. However, the therapeutic effects of agents targeting these receptors are limited or vary considerably among groups, indicating the involvement of an unknown pathological factor that modifies astrocyte and ApoE function. Here, we focused on the signaling lipid, sphingosine-1-phosphate (S1P), which is mainly produced by sphingosine kinase 2 (SphK2) in the brain. Using astrocyte models, we found that upregulation of SphK2/S1P signaling suppressed ApoE induction by both RXR and LXR agonists. We also found that SphK2 activation reduced RXR binding to the *APOE* promoter region in the nucleus, suggesting the nuclear function of SphK2/S1P. Intriguingly, suppression of SphK2 activity by RNA knockdown or specific inhibitors upregulated lipidated ApoE induction. Furthermore, the induced ApoE facilitates Aβ uptake in astrocytes. Together with our previous findings that SphK2 activity is upregulated in AD brain and promotes Aβ production in neurons, these results indicate that SphK2/S1P signaling is a promising multifunctional therapeutic target for AD that can modulate astrocyte function by stabilizing the effects of RXR and LXR agonists, and simultaneously regulate neuronal pathogenesis.

## Graphical abstract

**Figure:**
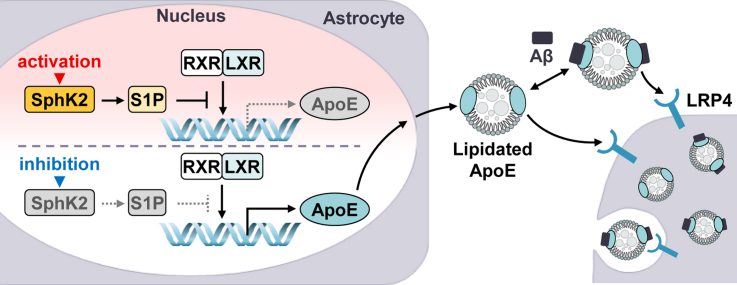

Alzheimer’s disease (AD) is a neurodegenerative disease characterized by extracellular accumulation of senile plaques composed mainly of amyloid β (Aβ), and intracellular neurofibrillary tangles composed of highly phosphorylated tau. Aβ accumulates in the brain prior to tau protein, and the imbalance between Aβ production and clearance is considered to be a cause of AD (1). Thus, Aβ-targeted therapeutics have been developed, and Aβ antibodies have received Food and Drug Administration approval for the treatment of AD. However, their efficacy in improving cognitive function is limited (2, 3), suggesting the need for comprehensive regulation of the brain environment, including Aβ.

Recently, neuroinflammation has attracted attention as the third hallmark of AD pathology (4). Neuroinflammation is characterized by the activation of glial cells, such as microglia and astrocytes, and impairs brain network function through the abnormal production of inflammatory cytokines. Notably, astrocyte activity is an executing factor (5). Astrocytes are the most abundant cell type in the brain and are responsible for maintaining lipid homeostasis (6). Astrocyte activation leading to a chronic inflammatory response has been proposed as a mechanism linking Aβ accumulation and tau pathology (7) and is considered to be one of the important pathological processes in AD. In addition, reactive astrocytes induced by microglial activation induce cell toxicity via the secretion of saturated lipids (5, 8). Therefore, alterations in astrocyte function and lipid regulatory mechanisms are highly relevant to the pathophysiology of AD.

Interestingly, apolipoprotein E (ApoE), a major mediator of lipid transport in the brain, is downregulated in astrocytes in AD (9, 10), suggesting the imbalance of lipid metabolism. Because ApoE is mainly produced in astrocytes and has anti-inflammatory and Aβ-metabolizing effects, its induction is a promising therapeutic target for AD. ApoE expression is coordinately regulated by the ligand-activated nuclear receptors, retinoid X receptor (RXR) and LXR. Indeed, the administration of RXR or LXR agonists reduces Aβ aggregation and restores cognitive function in AD mouse model (11, 12). However, the therapeutic effects of RXR agonists, especially bexarotene, varied widely in follow-up studies (13, 14, 15, 16), suggesting that there is an unknown pathological factor causing this variation, and that the regulatory mechanism of RXR-ApoE needs to be elucidated to develop effective AD treatments.

In this study, we focused on sphingosine-1-phosphate (S1P), a signaling lipid, which we have previously shown to be involved in Aβ production in neurons (17). In brain, S1P is mainly generated by phosphorylation of sphingosine by sphingosine kinase 2 (SphK2). Because the deficiency of SGPL1, an S1P-degrading enzyme, leads to induction of astrogliosis (18), elevated levels of S1P are considered an important activator of astrocyte. Interestingly, Aβ aggregation in AD model mice was reduced upon SphK2 KO (19), predicting a link between SphK2 activity and the disease state. Furthermore, because S1P is associated with transcriptional regulation to alter cellular function (20), we analyzed the relationship between SphK2/S1P signaling and ApoE using several astrocyte models.

## Material and Methods

### Reagents and antibodies

T0901317, bexarotene, ABC294640, SLM6031434, and FTY720 were purchased from Cayman Chemical (Ann Arbor, MI). Trichostatin A (#HY-15144) was purchased from MedChemExpress (Monmouth Junction, NJ). Synthetic Aβ_1-42_ peptide (Peptide Institute, Osaka, Japan) was solubilized in hexafluoro-2-propanol at a concentration of 1 mg/ml to prevent self-aggregation. For immunoblotting and immunostaining, antibodies against human Aβ N terminal (clone 82E1; Immuno-Biological Laboratories, Gunma, Japan), ATP binding cassette transporter A1 (ABCA1) (clone HJ1; Abnova, Taipei, Taiwan), ApoE (#AB947; Merck Millipore, Burlington, MA), α-tubulin (clone 10G10; Fujifilm Wako, Osaka, Japan), β-actin (#5125; Cell Signaling Technology, Danvers, MA), GAPDH (#2118; Cell Signaling Technology), glial fibrillary acidic protein (GFAP) (#GTX108711; GeneTex, Irvine, CA), Lamin A/C (#10298-1-AP; Proteintech, Rosemont, IL), LXRα/β (#sc-377260; Santa Cruz Biotechnology, Dallas, TX), nuclear factor-κB (NF-κB) p65 (#8242; Cell Signaling Technology), RXRα (#sc-515929; Santa Cruz Biotechnology), SphK2 (#17096-1-AP; Proteintech), and 6His (#66005-1-Ig; Proteintech) were purchased from the indicated vendors.

### Plasmids

pcDNA3.1-SphK2-V5-6His: The expression vector for human SphK2 conjugated with V5 and 6His tag in pcDNA3.1 vector was previously described (17).

pCAGGS-BLRP-hRXRα-IRES-BirA: pCAGGS-BLRP-IRES-BirA vector was purchased from RIKEN BRC (Ibaraki, Japan). The sequence of human RXRα was amplified from complementary DNA (cDNA) from U87 cells and inserted into pCAGGS-BLRP-IRES-BirA digested with NotI to express hRXRα N terminally fused to the biotin ligase recognition peptide (BLRP) sequence. To concatenate vector with DNA fragment, In-Fusion cloning system (TaKaRa Bio, Shiga, Japan) was used.

pFN21AA0816-LRP4-V5-6His: To express low-density lipoprotein receptor-related protein 4 (LRP4) fused with V5-6His tag at the C terminus, LRP4-Halo expression plasmid, pFN21AA0816, was purchased from Kazusa DNA Research Institute (Chiba, Japan). The vector was digested with NheI and AsiSI to eliminate the HaloTag sequence and replace the multi cloning site amplified from the pEBMulti-Hyg vector (Fujifilm Wako). The constructed vector was further cut with PmeI to insert the V5 and 6His tag sequence from the pcDNA3.1 vector. To concatenate vector with DNA fragment, NEBuilder HiFi DNA Assembly system (New England Biolabs, Ipswich, MA) was used.

pAAV2-CMV-SphK2: pAAV2-CMV vector was purchased from TaKaRa Bio. The DNA sequence encoding human SphK2-V5-6His was subcloned into the SpeI-digested pAAV2-CMV vector.

pAAV2-GfaABC_1_D-SphK2: The astrocyte-specific promoter, the GfaABC_1_D sequence (21) was synthesized by Thermo Fisher Scientific (Waltham, MA). pAAV2-CMV vector was digested with HindIII and EcoRI to remove the cytomegalovirus (CMV) promoter sequence and replace it with the GfaABC_1_D sequence. The DNA sequence encoding human SphK2 V5-6His tag-conjugate was subcloned into pAAV2-GfaABC_1_D vector digested with SpeI.

All constructed plasmids and inserted sequences were verified by Sanger sequencing (Azenta Life Sciences, Burlington, MA).

### Cell culture

Human astrocytoma U87 MG (U87) and human embryonic kidney (HEK) 293T cells were cultured in DMEM supplemented with 10% (v/v) heat-inactivated FBS and 1% penicillin/streptomycin at 37°C in a humidified atmosphere of CO_2_/95% air.

Human primary astrocytes (#CC-2565) derived from normal fetuses at 19 weeks of gestation were purchased from Lonza (Basel, Switzerland). Astrocytes were cultured in 1×ABM™ Basal Medium (#CC-3187; Lonza) containing 1×AGM™ SingleQuots™ Supplement Pack (#CC-4123; Lonza) [FBS, L-glutamine, GA-1000, ascorbic acid, hEGF, and insulin].

### Establishment of stable cell line

The pcDNA3.1-SphK2-V5-6His vector was introduced into U87 cells using Lipofectamine® 2000 (Thermo Fisher Scientific). Cells were selected with 500 μg/ml of Geneticin (G418).

### Immunoblotting

To detect ApoE and ABCA1, cells were preincubated with LXR or RXR agonist for 48 h in serum-free medium containing 1% penicillin/streptomycin and 0.1% BSA. To examine the effect of SphK2 on ApoE expression, cells were cotreated with SphK2 specific inhibitor or FTY720, and LXR or RXR agonist for 48 h.

Cell medium was mixed with 0.1 μM PMSF. After centrifugation (800 *g*, 4°C, 10 min), the supernatant was collected.

Cells were washed with ice-cold PBS and lysed with RIPA buffer [50 mM Tris–HCl (pH 7.5), 150 mM NaCl, 0.1% SDS, 1% Triton X-100, and 1% sodium deoxycholate containing protease inhibitor cocktail (Roche, Basel, Switzerland)] for 10 min on ice.

After BCA protein assay, the samples were boiled in SDS sample buffer [62.5 mM Tris–HCl (pH 6.8), 5% 2-mercaptoethanol, 2% SDS, and 10% glycerol], and separated by SDS-PAGE. The separated proteins were transferred to a 0.4 μm PVDF membrane. This membrane was blocked with nonfat milk in Tris-buffered saline containing 0.1% Tween-20 and incubated with appropriate primary antibodies at 4°C overnight. Then, HRP-conjugated secondary antibodies were applied to the membrane at room temperature for 1 h. Protein bands were visualized by the enhanced chemiluminescence system using the ChemiDoc™ MP Imaging System (Bio-Rad Laboratories, Hercules, CA). For the densitometric analysis, Image Lab software (Bio-Rad Laboratories; https://www.bio-rad.com/en-jp/product/image-lab-software?ID=KRE6P5E8Z) was used.

To detect Aβ_1-42_, Tris-Tricine SDS-PAGE was employed. The separated proteins were transferred to a 0.2 μm PVDF membrane (#BSP0161; PALL Life Science, Port Washington, NY). This membrane was boiled in PBS to expose the Aβ_1-42_ antigenic site before blocking with nonfat milk.

The same amount of protein was applied to each sample. The densitometric intensity of each blot was measured and normalized by the loading control indicated in the figure legends and used for statistical analysis.

### RT-PCR

Cells were washed with ice-cold PBS. Total RNA from U87 cells was extracted by the phenol–chloroform method using TRI reagent (#TR118; Molecular Research Center, Cincinnati, OH) and chloroform. ReverTra Ace (#TRT-101; TOYOBO, Osaka, Japan) was used to synthesize cDNA according to the manufacturer’s instructions. The PCR was performed with Expand High Fidelity^PLUS^ PCR System (Roche) under the following conditions. PCR products were separated on 2% agarose gels. DNA bands were visualized with 0.5 ng/ml ethidium bromide using the Gel Doc™ EZ System (Bio-Rad Laboratories). For analysis, Image Lab software was used, and the densitometric intensity was normalized by the level of actin beta (*ACTB*).

### Primer sequences

**Table:** 

Name	Primer Sequences	
*APOE*	Forward	5′- TGT TGG TCC CAT TGC TGA CAG GAT -3′
	Reverse	5′- TGG TGT TTA CCT CGT TGC GGT ACT -3′
*ABCA1*	Forward	5′- GCA CTG AGG AAG ATG CTG AAA -3′
	Reverse	5′- AGT TCC TGG AAG GTC TTG TTC AC -3′
*ACTB*	Forward	5′- CCT GAC GGC CAG GTC ATC -3′
	Reverse	5′- GGA CTC GTC ATA CTC CTG -3′

### Reverse transcription-quantitative PCR

Cells were washed with ice-cold PBS. Total RNA from U87 cells was extracted by the phenol–chloroform method using TRI reagent and chloroform. ReverTra Ace qPCR RT Master Mix (#FSQ-201; TOYOBO) was used to synthesize cDNA according to the manufacturer’s instructions. qPCR was performed using KOD SYBR qPCR Mix (#QKD-201; TOYOBO) under the following conditions: 98°C for 2 min, followed by 40 cycles of 98°C for 10 s, 60°C for 10 s, and 68°C for 30 s using CFX Duet (Bio-Rad Laboratories). All qPCRs amplified single products, as confirmed by the melting curve and electrophoresis using 2% agarose gels with 0.5 ng/ml ethidium bromide. The following primer sets were used. The 2^−ΔΔCt^ relative quantification method, using *ACTB* for normalization, was used to estimate target gene expression. The fold-change was calculated relative to the mRNA expression levels in the control samples.

### Primer sequences

**Table:** 

Name	Primer Sequences	
*APOE*	Forward	5′- GAG CAA GCG GTG GAG ACA G -3′
	Reverse	5′- CAT CAG CGC CCT CAG TTC C -3′
*ABCA1*	Forward	5′- GCA AGG CTA CCA GTT ACA TTT G -3′
	Reverse	5′- GTC AGA AAC ATC ACC TCC TG -3′
*APOJ*	Forward	5′- TCT TCT TTC CCA AGT CCC GC -3′
	Reverse	5′- TCA TCG TCG CCT TCT CGT ATG -3′
*IL6*	Forward	5′- ACT CAC CTC TTC AGA ACG AAT TG -3′
	Reverse	5′- CCA TCT TTG GAA GGT TCA GGT TG -3′
*IL1β*	Forward	5′- ATG ATG GCT TAT TAC AGT GGC AA -3′
	Reverse	5′- GTC GGA GAT TCG TAG CTG GA -3′
*ACTB*	Forward	5′- TCA CCC ACA CTG TGC CCA TCT ACG A -3′
	Reverse	5′- CAG CGG AAC CGC TCA TTG CCA CTG G -3′

### In vitro SphK2 activity assay

SphK2 activity assay was performed as previously described (17). Briefly, cells were washed with ice-cold PBS and lysed by freeze-thaw cycle in 50 mM HEPES (pH 7.4), 10 mM KCl, 15 mM MgCl_2_, 20% glycerol, 2 mM Na_3_VO_4_, 2 mM DTT, 10 mM NaF, 1 mM deoxypyridoxine, and EDTA-free complete protease inhibitor (Roche). Samples were centrifuged at 20,600 *g* for 10 min and the supernatants were collected. The lysates and 7-nitro-2,1,3-benzoxadiazole (NBD)-Sphingosine (Cayman Chemical) were mixed in the reaction buffer [50 mM HEPES (pH 7.4), 15 mM MgCl_2_, 0.5 M KCl, 10% glycerol, and 2 mM ATP] and incubated at 37°C for 30 min. The reactions were stopped by the addition of an equal volume of 1 M potassium phosphate (pH 8.5), followed by the addition of 2.5-times chloroform/methanol (2:1), and then centrifuged at 20,600 *g* for 1 min. The alkaline aqueous phase containing NBD-S1P, but not NBD-Sphingosine, was collected. After the aqueous phase combining with an equal volume of dimethylformamide, the fluorescence intensity was measured using the GloMax-Multi Detection System (Promega, Madison, WI).

### RNA interference knockdown

For SphK2 knockdown, the following siRNA targeting the SphK2 untranslated region (2639–2661) was purchased from Sigma-Aldrich (St. Louis, MO); (sense) ACA UUA GUG CAA AUC CUA GCG, (antisense) CUA GGA UUU GCA CUA AUG UUC. This siRNA sequence was introduced into U87 cells with Lipofectamine® 2000 or Lipofectamine™ RNAiMAX (Thermo Fisher Scientific).

For ApoE knockdown, the siRNA targeting ApoE was purchased from Sigma-Aldrich (#SASI_Hs01_00154592). This siRNA sequence was introduced into U87 cells with Lipofectamine™ RNAiMAX.

### Establishment of adeno-associated virus (AAV) vector and transduction

AAV was produced using the AAVpro Helper Free system (#6230; TaKaRa Bio) according to the manufacturer’s protocol. AAV particles were produced in HEK293T cells by cotransfection with pAAV2-CMV-SphK2, pRC2-mi342, and pHelper vector. Following day, the medium was changed to DMEM containing 2% FBS and 1% penicillin/streptomycin. At 48 h post transfection, AAV was extracted from AAV-producing HEK293T cells using AAV extraction solution (#6235; TaKaRa Bio). The final titers used in the experiments were 1.88 × 10^8^ copies/ml. Titers were determined by quantitative PCR using AAVpro® Titration Kit (for Real Time PCR) Ver.2 (#6233; TaKaRa Bio).

AAVs were transduced into human primary astrocytes for 48 h. Cell medium was mixed with 0.1 μM PMSF. After centrifugation (800 *g*, 4°C, 10 min), the supernatant was collected. Cells were washed with ice-cold PBS and lysed with RIPA buffer for 10 min on ice.

### Hippocampal slice culture

All animal experiments were performed in strict accordance with the institutional guidelines for animal experiments of Okayama University. The protocol was approved by the Animal Experimentation Committee of Okayama University (approval number: OKU-2022622). All animals were maintained in an environment at 24°C with a 12 h light/dark cycle, with water and food freely available.

C57BL/6J mice (postnatal days 7–10) were obtained from CLEA Japan (Tokyo, Japan). Hippocampal slice culture was performed as previously described (22, 23). For slice culture, adult mice were euthanized by decapitation after pentobarbital administration. Pups were euthanized by decapitation under hypothermic anesthesia. Mouse brains were sectioned into 400 μm thick slices using a tissue chopper [McIlwain] with a cutting blade. The hippocampal slices were dissected out and incubated at 4°C for 60 min in incubation medium containing minimum essential medium, 9 mM Tris, 22.9 mM HEPES, and 63.1 mM glucose supplemented with penicillin/streptomycin. The slices were placed on Omnipore® membrane filters (#JHWP02500; Merck Millipore) on the doughnut plate (Hazai-Ya, Tokyo, Japan) in a solution containing 50% minimum essential medium, 25% HBSS, 25% horse serum (#H1138; Sigma-Aldrich), 6.6 mM Tris, 16.9 mM HEPES, 4 mM NaHCO_3_, and 29.8 mM glucose. The prepared slices were maintained at 37°C with 5% CO_2_/95% air. The culture medium was replaced twice per week.

For ApoE induction, the serum concentration was gradually decreased from 4 to 6 days in vitro (DIV) and replaced with B-27 (#17504044; Thermo Fisher Scientific) -supplemented Neurobasal-A medium (#10888022; Thermo Fisher Scientific).

### Establishment of astrocyte-specific AAV vector and transduction

AAV was produced using the AAVpro Helper Free system according to the manufacturer’s protocol. AAV particles were produced in HEK293T cells by cotransfection with pAAV2-GfaABC_1_D-SphK2, pRC2-mi342, and pHelper vector. Following day, the medium was changed to DMEM containing 1% penicillin/streptomycin. At 48 h post transfection, AAV was extracted from AAV-producing HEK293T cells using AAV extraction solution. The final titers used in the experiments were 2.19 × 10^8^ copies/ml. Titers were determined by quantitative PCR using AAVpro® Titration Kit (for Real Time PCR) Ver.2. AAVs were transduced into hippocampal slices at DIV1.

### Immunostaining for cultured hippocampal slice

For immunostaining, the cultured slices were fixed with 4% paraformaldehyde in PBS at 4°C for 1 h. The fixed slices were washed with PBS and permeabilized with PBS containing 1% Triton X-100 and 5% horse serum at room temperature for 1 h. Slices were incubated with primary antibodies against GFAP (1:500) and 6His (1:200) at 4°C for 48 h. Slices were washed three times with PBS and incubated with secondary antibodies conjugated with Alexa Fluor 488 or 594 (1:500) at 4°C overnight. Slices were washed three times with PBS and then treated with VECTASHIELD Vibrance Antifade Mounting Medium with 4',6-diamidino-2-phenylindole (DAPI) (#H-1800; Vector Laboratories, Newark, CA). Images were obtained using BZ-X800 (Keyence, Osaka, Japan).

### Immunoblotting for cultured hippocampal slice

To detect ApoE, slices were preincubated with RXR agonist for 48 or 96 h in serum-free condition.

The medium was mixed with 0.1 μM PMSF. After centrifugation (800 *g*, 4°C, 10 min), the supernatant was collected.

Cultured hippocampal slices were washed with ice-cold PBS and lysed with RIPA buffer [25 mM Tris–HCl (pH 7.5), 137 mM NaCl, 3 mM KCl, 0.1% SDS, 1% NP-40, and 0.5% sodium deoxycholate containing protease inhibitor cocktail] (24). After BCA protein assay, the samples were boiled in SDS sample buffer and separated by SDS-PAGE.

### Nuclear extraction

Nuclear extraction was performed using Nuclear Extract Kit (#40010; Active Motif, Carlsbad, CA). The cell medium was removed, and the cells were collected in phosphatase/PBS using a cell lifter. After centrifugation (200 *g*, 4°C, 5 min), the cell pellet was suspended in hypotonic buffer and detergent. This suspension was centrifuged (14,000 *g*, 4°C, 30 s) and the supernatant was collected as the cytoplasmic fraction. The pellet was resuspended in lysis buffer and detergent. After centrifugation (14,000 *g*, 4°C, 10 min), the supernatant was collected as the nuclear fraction.

### Chromatin immunoprecipitation (ChIP)

U87 cells were transfected with pCAGGS-BLRP-hRXRα-IRES-BirA using Lipofectamine® 3000 (Thermo Fisher Scientific). The medium was changed to serum-free medium containing 1% penicillin/streptomycin, 0.1% BSA, and 50 μg/ml D-biotin 24 h after the transfection. Following day, the cells were treated with bexarotene for 3 h.

DNA and protein were cross-linked with 1% formaldehyde at room temperature for 10 min. Then, 0.15 M glycine was added to quench cross-linking. Fixed cells were washed with the buffer containing PBS and 2% FBS and lysed with SDS lysis buffer [50 mM Tris–HCl (pH 8.0), 10 mM EDTA (pH 8.0), and 1% SDS containing protease inhibitor cocktail]. The cells were then sonicated with 30 s pulses (output: 6) for 5 cycles, with a 1 min interval using a sonicator (#VP-5s; TAITEC, Saitama, Japan) and centrifuged (20,600 *g*, 8°C, 10 min). The supernatant was diluted in ChIP dilution buffer [50 mM Tris–HCl (pH 8.0), 167 mM NaCl, 1.1% Triton X-100, and 0.11% sodium deoxycholate containing protease inhibitor cocktail], and one-tenth samples were collected as input samples.

For the pull-down, NeutrAvidin™ Agarose (#29220; Thermo Fisher Scientific) was washed twice with RIPA buffer for ChIP [50 mM Tris–HCl (pH 8.0), 150 mM NaCl, 1 mM EDTA, 1% Triton X-100, 0.1% SDS, and 0.1% sodium deoxycholate], and resuspended with salmon sperm DNA in RIPA buffer to give a 50% slurry. The beads were mixed with the ChIP sample and rotated at 4°C overnight. The beads were then washed with RIPA buffer containing 150 mM or 500 mM NaCl, LiCl wash buffer [10 mM Tris–HCl (pH 8.0), 0.25 M LiCl, 1 mM EDTA, 0.5% NP-40, and 0.5% sodium deoxycholate], and Tris-EDTA buffer. After washing, the beads were eluted with ChIP elution buffer [10 mM Tris–HCl (pH 8.0), 300 mM NaCl, 5 mM EDTA, and 0.5% SDS]. DNA–protein crosslinks were reversed at 65°C for 4 h, then treated with RNase and proteinase K at 37°C for 30 min and at 55°C for 1 h, respectively. DNA was purified by phenol/chloroform extraction and eluted with Tris-EDTA buffer. Finally, the target DNA was amplified by PCR. The following primers were designed against the LXR response element region in *APOE* promoter (25). The sequence specificity was confirmed by GGGenome (https://gggenome.dbcls.jp/ja/hg19/).

### Primer sequences

**Table:** 

Name	Primer Sequences	
*APOE* LXRE	Forward	5′- TTT TGT ATT TTT AGT AGA GAT GGG GTT T -3′
	Reverse	5′- AGT AAT ACA GAC ACC CTC CTC CAT T -3′

### Native-PAGE

Cell medium was collected and mixed with 0.1 μM PMSF. After centrifugation (800 *g*, 4°C, 10 min), the supernatant was collected. Sample buffer [200 mM Tris–HCl (pH 6.8), 40% glycerol, and 0.04% Coomassie brilliant blue R-250] was applied to the sample.

Polyacrylamide gel without SDS was used and kept at 4°C during electrophoresis. The separated proteins were transferred to a 0.4 μm PVDF membrane at 4°C for 15 h 20 min. This membrane was blocked with nonfat milk in Tris-buffered saline containing 0.1% Tween-20 and incubated with appropriate primary antibodies at 4°C overnight. Then, HRP-conjugated secondary antibodies were applied to the membrane at room temperature for 1 h. Protein bands were visualized by the enhanced chemiluminescence system using the ChemiDoc™ MP Imaging System. For the densitometric analysis, Image Lab software was used.

### Coimmunoprecipitation using anti-Aβ antibody (4G8)

U87 cells were treated with RXR agonist or SphK2 inhibitor for 48 h. Cell medium was collected and mixed with 0.1 μM PMSF. After centrifugation (800 *g*, 4°C, 10 min), the supernatant was collected as conditioned medium. The solvent for synthetic Aβ_1-42_ was replaced from hexafluoro-2-propanol to DMSO immediately before use. Synthetic Aβ_1-42_ peptide was applied to the conditioned medium at a final concentration of 1 μM and rotated at 4°C overnight. Protein A/G PLUS Agarose (#sc-2003; Santa Cruz Biotechnology) was blocked with 1% BSA/PBS for 1 h and equilibrated with equivalent serum-free medium containing 1% penicillin/streptomycin. Equilibrated beads were mixed with U87 conditioned medium containing 1 μM Aβ_1-42_, anti-β-Amyloid 17–24 antibody (clone 4G8; BioLegend, San Diego, CA) and Triton X-100, and rotated at 4°C overnight. After centrifugation (1,500 *g*, 4°C, 3 min), 4G8 wash buffer [50 mM NaCl, 10 mM Tris–HCl (pH 7.6), and 0.01% Triton X-100] was applied to the mixture and rotated at 4°C for 20 min. After additional centrifugation (1,500 *g*, 4°C, 3 min), 4G8 wash buffer was added to the pellet and resuspended. The mixture was layered on 1 M sucrose/4G8 wash buffer and centrifuged (9,200 *g*, 4°C, 1 min). Beads were washed with Tris–HCl (pH 7.6) and centrifuged (1,500 *g*, 4°C, 3 min). Immunoprecipitation samples were boiled in 2× SDS sample buffer immediately before use.

#### Transferrin uptake

Reverse-transfected U87 cells were seeded in 12 well plates on poly L-lysine-coated slides. Following day, the medium was replaced with DMEM supplemented with 0.1% BSA without serum and the cells were incubated for 1 h. The cells were then washed twice with PBS and replaced with medium containing Fluorescein (FITC)-conjugated ChromPure Human Transferrin (Jackson Immuno Research Laboratories, Inc., West Grove, PA) diluted to 10 μg/ml and incubated at 37°C for 30 min. The cells were preincubated at 4°C for 10 min, and fluorescent transferrin was added in the same manner and incubated at 4°C for 30 min to serve as a control. Cells were washed twice with ice-cold PBS and fixed in PBS containing 4% paraformaldehyde for 30 min. The cells were then incubated with PBS containing 1 μg/ml DAPI for 10 min, washed twice with PBS, sealed, and observed using BZ-X800.

### Aβ uptake assay

U87 cells were seeded for the reagent treatment or transfection. One group of cells was treated with reagents for 48 h, and the conditioned medium was collected. The other group of cells was transiently transfected with pFN21AA0816-LRP4-V5-6His vector using PEI MAX (Polysciences, Warrington, PA) and after 24 h, the medium was changed to serum-free medium containing 1% penicillin/streptomycin and 0.1% BSA. The solvent for synthetic Aβ_1-42_ was replaced from hexafluoro-2-propanol to DMSO immediately before use. Synthetic Aβ_1-42_ peptide was applied to the conditioned medium at a final concentration of 0.2 μM and rotated at 4°C overnight. Then, this medium and lysosome inhibitor, chloroquine (10 μM), were applied to LRP4-expressing U87 cells. Cell medium was centrifuged (800 *g*, 4°C, 10 min), and the supernatant was collected. Cells were washed with ice-cold PBS and acid wash solution [0.2 M acetic acid and 0.5 M NaCl] for 6 min and lysed with RIPA buffer. After BCA protein assay, cell medium or lysate samples were boiled in SDS sample buffer and applied to immunoblotting. The densitometric intensity of intracellular ApoE and Aβ was measured as the amount of uptake using Image Lab software.

### Statistical analysis

All experiments were independently repeated at least three times. All data represent mean ± SEM. Statistical analysis was performed by two-tailed Student’s *t-*test or one-way ANOVA with Dunnett’s or Bonferroni’s post *hoc test* in GraphPad Prism 8 (GraphPad Software, San Diego, CA; https://www.graphpad.com/features). Significance was set at ∗*P* < 0.05, ∗∗*P* < 0.01, ∗∗∗*P* < 0.001, ∗∗∗∗*P* < 0.0001.

## Results

### SphK2 activation inhibits ApoE induction by LXR/RXR agonists, but not ABCA1

**Fig. 1:**
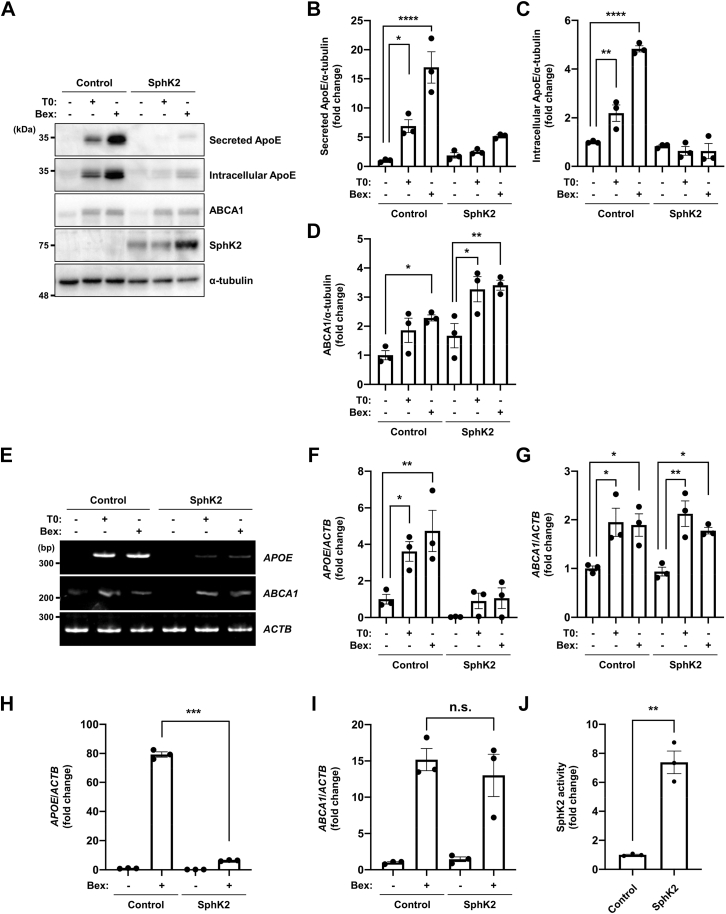
ApoE induction by LXR/RXR agonists is inhibited in U87-SphK2 cells. A: Immunoblotting analysis for ApoE, ABCA1, and SphK2 in U87-control and U87-SphK2 cells. Cells were incubated with serum-free medium and treated with 1 μM T0901317 (T0) or 1 μM bexarotene (Bex) for 48 h. Medium was changed to serum-free 2 h after cell seeding. B and C: Quantification of secreted (B) and intracellular (C) ApoE in U87-control and U87-SphK2 cells shown in Figure 1A. The relative protein levels were normalized to those of α-tubulin. Data are expressed as mean ± SEM (*n* = 3, ∗*P* < 0.05, ∗∗*P* < 0.01, ∗∗∗∗*P* < 0.0001: significant difference compared with the vehicle sample in U87-control cells by one-way ANOVA with Dunnett’s *post hoc* test). D: Quantification of ABCA1 in U87-control and U87-SphK2 cells shown in Figure 1A. The relative protein levels were normalized to those of α-tubulin. Data are expressed as mean ± SEM (*n* = 3, ∗*P* < 0.05, ∗∗*P* < 0.01: significant difference compared with the vehicle sample in U87-control or U87-SphK2 cells by one-way ANOVA with Bonferroni’s *post hoc* test). E: RT-PCR analysis for *APOE* and *ABCA1* in U87-control and U87-SphK2 cells. Cells were incubated with serum-free medium and treated with 1 μM T0901317 or 1 μM bexarotene for 48 h. Medium was changed to serum-free 2 h after cell seeding. F: Quantification of *APOE* mRNA in U87-control and U87-SphK2 cells shown in Figure 1E. The relative mRNA levels were normalized to those of *ACTB*. Data are expressed as mean  ±  SEM (*n* = 3, ∗*P* < 0.05, ∗∗*P* < 0.01: significant difference compared with the vehicle sample in U87-control cells by one-way ANOVA with Dunnett’s *post hoc* test). G: Quantification of *ABCA1* mRNA in U87-control and U87-SphK2 cells shown in Figure 1E. The relative mRNA levels were normalized to those of *ACTB*. Data are expressed as mean  ±  SEM (*n* = 3, ∗*P* <  0.05, ∗∗*P* < 0.01: significant difference compared with the vehicle sample in U87-control or U87-SphK2 cells by one-way ANOVA with Bonferroni’s *post hoc* test). H and I: Quantification of *APOE* (H) and *ABCA1* (I) mRNA in U87-control and U87-SphK2 cells by RT-qPCR analysis. Cells were incubated with serum-free medium and treated with 1 μM bexarotene for 48 h. Medium was changed to serum-free 2 h after cell seeding. The relative mRNA levels were normalized to those of *ACTB*. Data are expressed as mean  ±  SEM (*n* = 3, ∗∗∗*P* < 0.001: significant difference compared with the bexarotene treatment sample in U87-control cells by one-way ANOVA with Dunnett’s *post hoc* test). J: In vitro SphK2 activity in cell lysate. Cells were incubated for 48 h and lysates were collected. The lysates and NBD-sphingosine were mixed, and the alkaline aqueous phase containing NBD-S1P was collected using chloroform/methanol. The NBD-S1P-derived fluorescence was evaluated. Data are expressed as mean  ±  SEM (*n* = 3, ∗∗*P* < 0.01: significant difference compared with the control by two-tailed Student’s *t*-test). ABCA1, ATP binding cassette transporter A1; ACTB, actin beta; ApoE, apolipoprotein E; NBD, 7-nitro-2,1,3-benzoxadiazole; RT-qPCR, reverse transcription-quantitative PCR; RXR, retinoid X receptor; S1P, sphingosine-1-phosphate; SphK2, sphingosine kinase 2.

To analyze the physiological function of ApoE and its relationship with SphK2/S1P signaling in astrocytes, we established SphK2 stably overexpressing cells based on human astrocytoma U87 cell line (U87-SphK2 cells), which have the most common genotype of ApoE, ε3/ε3 (26). We used LXR agonist (T0901317) and RXR agonist (bexarotene) to induce ApoE production. Both agonists induced the expression of ApoE and ABCA1, which mediate the lipidation and secretion of ApoE as a lipoprotein. Interestingly, in U87-SphK2 cells, both agonists induced ABCA1 expression, similar to that in U87-control cells, whereas ApoE induction was strongly suppressed (Fig. 1A–D). We also found that both agonists upregulated the transcription of *APOE* and *ABCA1* in the U87-control cells. However, in U87-SphK2 cells, both agonists induced *ABCA1*, but failed to induce *APOE* transcription (Fig. 1E–I). The SphK2 activity was increased in U87-SphK2 cells (Fig. 1J), indicating that SphK2 activity plays a major role in regulating ApoE expression.

### Endogenous SphK2 activity regulates ApoE expression

**Fig. 2:**
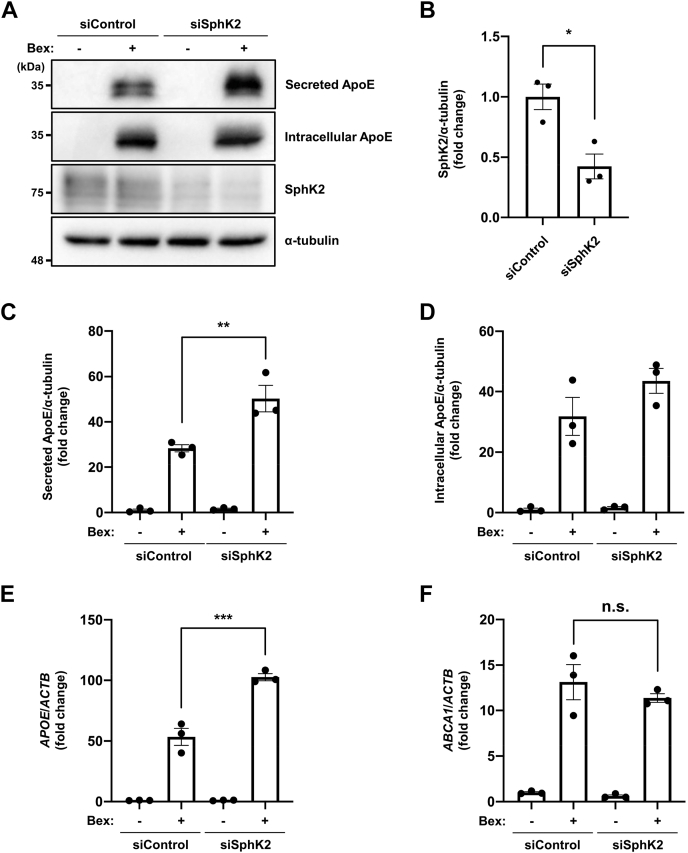
SphK2 knockdown increases ApoE induction by RXR agonist. A: Immunoblotting analysis for ApoE and SphK2 in control and SphK2 siRNA treated cells. U87-control cells were transfected with siRNA and after 24 h, treated with 1 μM bexarotene for 48 h. Medium was changed to serum-free 6 h after transfection. B: Quantification of SphK2 knockdown efficiency shown in Figure 2A. The relative protein levels were normalized to those of α-tubulin. Data are expressed as mean ± SEM (*n* = 3, ∗*P* < 0.05: significant difference compared with the control sample by two-tailed Student’s *t*-test). C and D: Quantification of secreted (C) and intracellular (D) ApoE in control and SphK2 siRNA treated cells shown in Figure 2A. The relative protein levels were normalized to those of α-tubulin. Data are expressed as mean ± SEM (*n* = 3, ∗∗*P* < 0.01: significant difference compared with the control sample treated with bexarotene by one-way ANOVA with Dunnett’s *post hoc* test). E and F: Quantification of *APOE* (E) and *ABCA1* (F) mRNA in control and SphK2 siRNA treated cells by RT-qPCR analysis. U87-control cells were transfected with siRNA and after 24 h, treated with 1 μM bexarotene for 48 h. Medium was changed to serum-free 6 h after transfection. The relative mRNA levels were normalized to those of *ACTB*. Data are expressed as mean  ±  SEM (*n* = 3, ∗∗∗*P* < 0.001: significant difference compared with the control sample treated with bexarotene by one-way ANOVA with Dunnett’s *post hoc* test). ABCA1, ATP binding cassette transporter A1; ACTB, actin beta; ApoE, apolipoprotein E; RT-qPCR, reverse transcription-quantitative PCR; RXR, retinoid X receptor; SphK2, sphingosine kinase 2.

**Fig. 3:**
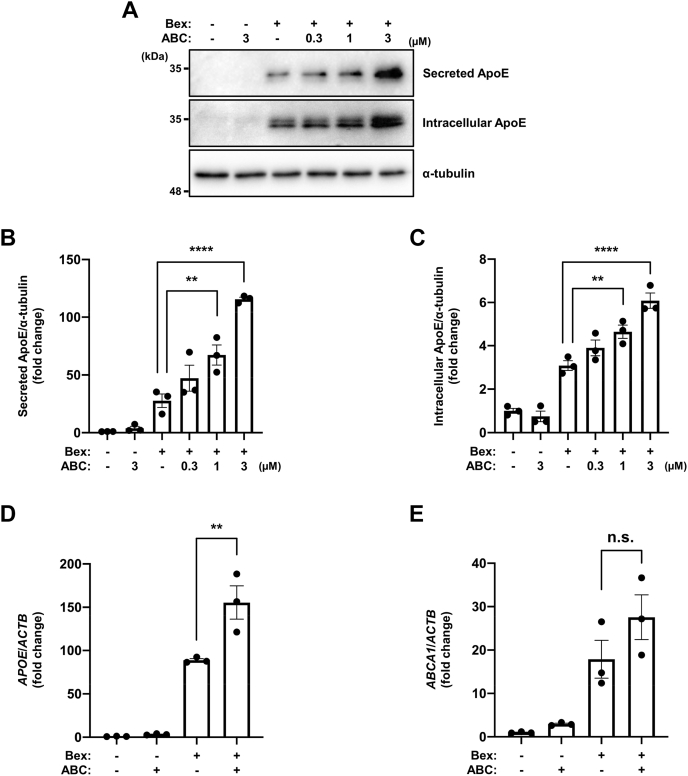
SphK2 inhibitor increases ApoE induction by RXR agonist. A: Immunoblotting analysis for ApoE under the SphK2 inhibition condition. U87-control cells were incubated with serum-free medium and treated with 1 μM bexarotene and the indicated concentration of ABC294640 (ABC) for 48 h. Medium was changed to serum-free 2 h after cell seeding. B and C: Quantification of secreted (B) and intracellular (C) ApoE shown in Figure 3A. The relative protein levels were normalized to those of α-tubulin. Data are expressed as mean ± SEM (*n* = 3, ∗∗*P* < 0.01, ∗∗∗∗*P* < 0.0001: significant difference compared with the bexarotene treatment sample by one-way ANOVA with Dunnett’s *post hoc* test). D and E: Quantification of *APOE* (*D*) and *ABCA1* (*E*) mRNA under the SphK2 inhibition condition by RT-qPCR analysis. U87-control cells were incubated with serum-free medium and treated with 1 μM bexarotene and the indicated concentration of ABC294640 for 48 h. Medium was changed to serum-free 2 h after cell seeding. The relative mRNA levels were normalized to those of *ACTB*. Data are expressed as mean ± SEM (*n* = 3, ∗∗*P* < 0.01: significant difference compared with the bexarotene treatment sample by one-way ANOVA with Dunnett’s *post hoc* test). ABCA1, ATP binding cassette transporter A1; ACTB, actin beta; ApoE, apolipoprotein E; RT-qPCR, reverse transcription-quantitative PCR; RXR, retinoid X receptor; SphK2, sphingosine kinase 2.

To analyze the function of endogenous SphK2, we performed SphK2 knockdown using RNA interference and observed an approximately 60% reduction in SphK2 expression (Fig. 2A, B). In contrast to SphK2 overexpression, SphK2 knockdown resulted in enhanced induction of secreted ApoE by bexarotene (Fig. 2A, C, D), and of secreted and intracellular ApoE by T0901317 (supplemental Fig. S1A–C). In addition, SphK2 knockdown enhanced *APOE* expression (1.9-fold), but not *ABCA1* at the transcriptional level (Fig. 2E, F). ApoJ (clusterin), like ApoE, is an apolipoprotein abundant in astrocytes. Interestingly, *APOJ* transcription induced by RXR agonist was not affected by SphK2 overexpression or knockdown, suggesting that the effects of SphK2/S1P signaling are ApoE-specific in apolipoproteins (supplemental Fig. S1D, E). To further clarify its correlation with SphK2 activity, we cotreated the cells with bexarotene and SphK2 specific inhibitor, ABC294640 (27). Cotreatment with bexarotene and ABC294640 enhanced the induction of secreted (+1 μM ABC: 2.4-fold, +3 μM ABC: 4.1-fold) and intracellular (+1 μM ABC: 1.5-fold, +3 μM ABC: 1.9-fold) ApoE in a dose-dependent manner (Fig. 3A–C). *APOE* mRNA expression was also increased (+ABC: 1.8-fold) but not *ABCA1* by ABC294640 (Fig. 3D, E). In subsequent experiments, we chose the 3 μM treatment, which showed a marked increase in ApoE expression, to monitor the effect of ABC294640. In concordance with our finding, another SphK2 specific inhibitor, SLM6031434 (28), also enhanced the induction of ApoE by bexarotene (+0.1 μM SLM: 1.1-fold, +0.3 μM SLM: 1.2-fold, supplemental Fig. S2A–C). Our data revealed that the transcriptional regulation of ApoE and ABCA1 has distinct aspects and that SphK2 activity plays a key role in ApoE expression.

### Effect of SphK2/S1P signaling on ApoE expression in various astrocyte models

**Fig. 4:**
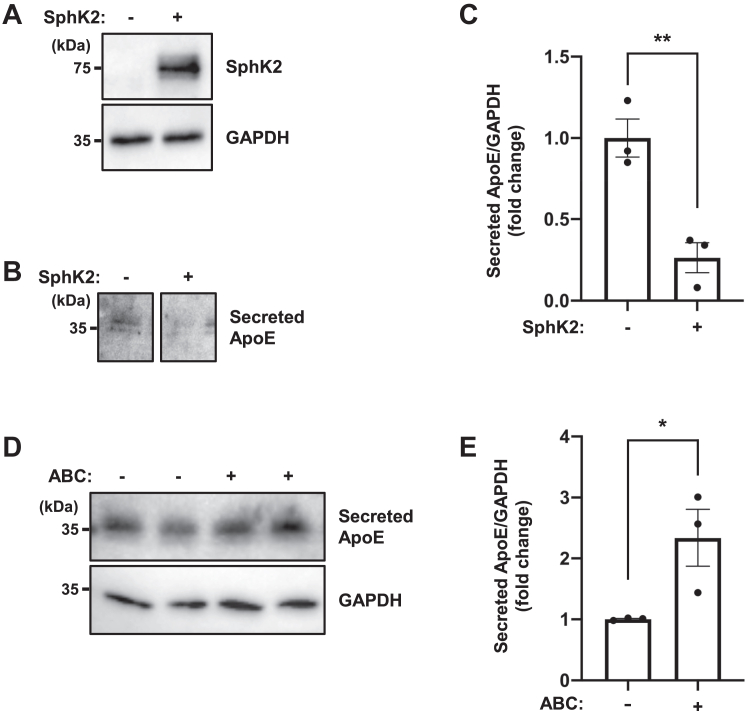
SphK2/S1P signaling regulates ApoE expression in human primary astrocytes. A and B: Immunoblotting analysis for SphK2 (A) and secreted ApoE (B). Human primary astrocytes were transduced with AAV2-CMV-SphK2. Following day, the medium was changed to serum-free and incubated for 48 h. C: Quantification of secreted ApoE shown in Figure 4B. The relative protein levels were normalized to those of GAPDH. Data are expressed as mean ± SEM (*n* = 3, ∗∗*P* < 0.01: significant difference compared with the control sample by two-tailed Student’s *t*-test). D: Immunoblotting analysis for secreted ApoE. Human primary astrocytes were treated with 3 μM ABC294640 for 48 h in serum-free condition. E: Quantification of secreted ApoE shown in Figure 4D. The relative protein levels were normalized to those of GAPDH. Data are expressed as mean  ±  SEM (*n* = 3, ∗*P* < 0.05: significant difference compared with the vehicle sample by two-tailed Student’s *t*-test). AAV, adeno-associated virus; ApoE, apolipoprotein E; ApoE, apolipoprotein E; CMV, cytomegalovirus; S1P, sphingosine-1-phosphate; SphK2, sphingosine kinase 2.

We used human primary astrocytes as another astrocyte model. First, an AAV vector expressing SphK2 under the control of the CMV promoter was transduced, and SphK2 overexpression decreased basal ApoE secretion (0.26-fold) (Fig. 4A–C). We also observed that ApoE secretion was significantly increased by treatment with the SphK2 inhibitor ABC294640 (+ABC: 2.3-fold) (Fig. 4D, E), suggesting that this mechanism is common in astrocytes.

**Fig. 5:**
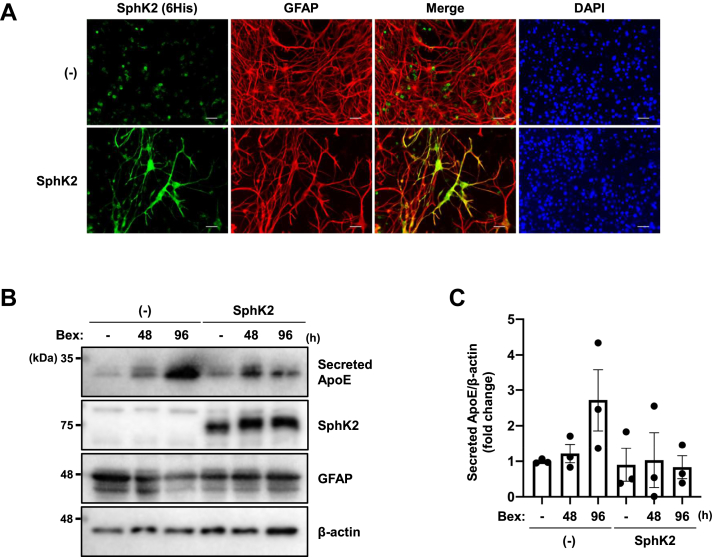
SphK2/S1P signaling tends to suppress ApoE expression in hippocampal slices. A: Immunostaining of hippocampal slice. V5-6His tagged SphK2 (green) and astrocytic marker GFAP (red) were detected. DAPI stained the nucleus (blue). Slices were incubated with serum-free medium consisting of MEM, HBSS, and B-27-supplemented Neurobasal-A and analyzed at DIV11. Scale bars represent 30 μm. B: Immunoblotting analysis for ApoE, SphK2, and GFAP in hippocampal slices. Slices were transduced with AAV2-GfaABC_1_D-SphK2 at DIV1. The slices were incubated with serum-free medium consisting of MEM, HBSS, and B-27-supplemented Neurobasal-A. At DIV7, the slices were treated with DMSO for 96 h or 1 μM bexarotene for 48 and 96 h. The medium was gradually changed to serum-free from DIV4 to 6. C: Quantification of secreted ApoE shown in Figure 5B. The relative protein levels were normalized to those of β-actin. Data are expressed as mean  ±  SEM (*n* = 3, significant difference compared with the vehicle sample by one-way ANOVA with Dunnett’s *post hoc* test). AAV, adeno-associated virus; ApoE, apolipoprotein E; DAPI, 4',6-diamidino-2-phenylindole; DIV, days in vitro; GFAP, glial fibrillary acidic protein; HBSS, Hanks’ balanced salt solution; MEM, minimum essential medium; S1P, sphingosine-1-phosphate; SphK2, sphingosine kinase 2.

Astrocytes survive and function in the brain through a complex network of other cells, such as neurons and microglia. We examined whether the phenomena observed in U87 cells could be reproduced in a hippocampal slice culture system in which neural circuits are maintained and neurons and glial cells coexist. Furthermore, hippocampal slice cultures have the advantage of easy gene transfer and drug treatment compared with animal models. Hippocampal slices were prepared from 400 μm thick sections of postnatal 7–10 days C57BL/6J mice. To specifically express SphK2 in astrocytes, an AAV-GfaABC_1_D-SphK2 vector was generated to express SphK2 under the GfaABC_1_D promoter, which is a truncated GFAP promoter. The GfaABC_1_D promoter has the transcriptional activity similar to that of the GFAP promoter (29). We confirmed that despite the presence of background fluorescence, the staining of expressed SphK2 was relatively consistent with GFAP, an astrocyte marker (Fig. 5A). Also, z-stack images indicate that SphK2 is expressed in the nucleus (supplemental Fig. S3). Hippocampal slices were transduced with AAV-GfaABC_1_D-SphK2 at DIV1. In addition, the serum concentration was gradually reduced and replaced with B-27-supplemented Neurobasal-A medium from DIV4 to DIV6 to reduce the reactivity of astrocytes and better simulate in vivo conditions (30). Hippocampal slices were treated with bexarotene and collected at 48 and 96 h post treatment. Although no statistically significant differences were obtained, hippocampal slices overexpressing SphK2 showed a trend toward reduced ApoE induction by bexarotene compared to control slices (Fig. 5B, C). These results suggest that SphK2/S1P signaling regulates ApoE expression in astrocytes and that this mechanism is conserved across species.

### FTY720 inhibits ApoE induction

The next question is whether S1P, a product of SphK2, is a bona fide mediator of the suppression of ApoE expression. Considering that S1P has low cell permeability (31) and is highly unstable (32), we analyzed FTY720, a synthetic analog of sphingosine. FTY720 localizes to the cytoplasm and nucleus after permeabilization of the plasma membrane, and is converted to its S1P analog, FTY720 phosphate (FTY720-P), upon phosphorylation by SphK2 (33). Interestingly, cotreatment with FTY720 and T0901317 markedly reduced the secreted (+1 μM FTY: 0.65-fold) and intracellular (+1 μM FTY: 0.55-fold) ApoE levels (supplemental Fig. S4A–C).

### SphK2/S1P signaling regulates astrocyte reactivity

Suppression of ApoE expression by SphK2/S1P signaling suggests the possibility of altered astrocyte function. As SphK2/S1P signaling involves in inflammatory responses in multiple cell types, we analyzed the changes in the activity of the NF-κB pathway and the expression of inflammatory cytokines. The subunit p65 of NF-κB was upregulated in U87-SphK2 cells and its translocation to the nucleus was increased (supplemental Fig. S5A). Moreover, interleukin 6 (*IL6*) and *IL1β* mRNA were upregulated in U87-SphK2 cells and SphK2 knockdown decreased their expression (supplemental Fig. S5B–E). These data suggest that SphK2/S1P signaling exacerbates inflammatory responses in astrocytes. To clarify whether the change in inflammatory status was due to ApoE, we examined basal ApoE levels based on Figure 1H data and found that *APOE* levels were significantly decreased in U87-SphK2 cells (supplemental Fig. S5F). Interestingly, the expression of *IL6* was significantly increased when ApoE was knocked down (supplemental Fig. S5G, H) suggesting that the basal secretion of ApoE has an anti-inflammatory role in astrocytes.

### Nuclear SphK2/S1P signaling regulates RXRα affinity to the target region and its expression

**Fig. 6:**
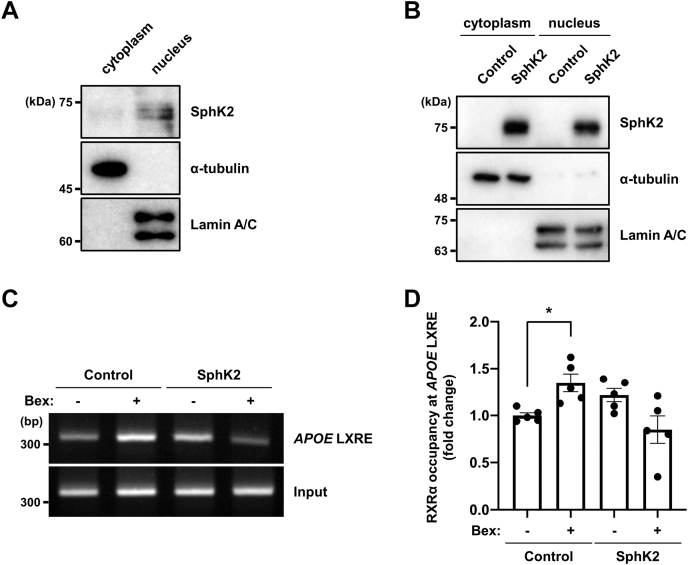
Nuclear SphK2/S1P signaling alters RXRα affinity to the *APOE* promoter. A and B: Nuclear fractionation analysis for SphK2. Endogenous (A) and expressed (B) SphK2 were detected in U87 cells. α-tubulin and Lamin A/C were used as cytoplasm and nuclear marker protein, respectively. C: ChIP analysis for RXRα binding to the *APOE* promoter region. U87-control and U87-SphK2 cells were transfected with pCAGGS-BLRP-hRXRα-IRES-BirA and after 24 h, treated with 50 μg/ml D-biotin along with medium change to serum-free condition. Following day, cells were treated with 1 μM bexarotene for 3 h. D: Quantification of RXRα occupancy at the *APOE* promoter region shown in Figure 6C. The relative levels of the enrichment of RXRα on the *APOE* promoter were normalized to those of input. Data are expressed as mean ± SEM (*n* = 5, ∗*P* < 0.05: significant difference compared with the vehicle sample in U87-control cells by one-way ANOVA with Dunnett’s *post hoc* test). ApoE, apolipoprotein E; BLRP, biotin ligase recognition peptide; ChIP, chromatin immunoprecipitation; LXRE, LXR response element; RXR, retinoid X receptor; S1P, sphingosine-1-phosphate; SphK2, sphingosine kinase 2.

Recently, the nuclear functions of S1P and FTY720-P have attracted attention (34, 35). Interestingly, the nuclear localization of SphK2 is increased in AD brains (36), suggesting a nuclear function of S1P in AD pathogenesis. Nuclear fractionation analysis showed that endogenous and expressed SphK2 is predominantly localized in the nucleus (Fig. 6A, B). Next, we performed a modified ChIP experiment utilizing biotin-avidin conjugation, which allowed for more specific analysis than the antigen–antibody reaction (37). We used the pCAGGS-BLRP-hRXRα-IRES-BirA vector, in which human RXRα was expressed with BLRP at the N terminus, and biotin is recruited to the BLRP by the coexpressed biotin ligase, BirA. U87 cells were transfected with BLRP-hRXRα and after 24 h treated with biotin. The following day, cells were treated with bexarotene for 3 h. The pull-down efficiency of this analysis was confirmed (supplemental Fig. S6). As previously reported (38), bexarotene treatment induced RXRα binding to the target region, around the LXR response element sequence in the *APOE* promoter region in U87-control cells (+bexarotene: 1.35-fold). Interestingly, in U87-SphK2 cells, bexarotene treatment failed to promote RXRα binding to the target region in the *APOE* promoter (Fig. 6C, D). This evidence indicates that nuclear SphK2/S1P functions as a modulator that inhibits RXRα binding to the *APOE* promoter region.

Because S1P and FTY720-P have been reported to function as inhibitors of histone deacetylases (HDAC) (34, 35), we examined the effect of the class I/II HDAC inhibitor, trichostatin A, on ApoE expression. Cotreatment with trichostatin A did not significantly affect ApoE induction by bexarotene (supplemental Fig. S7A, B), suggesting that HDAC activity is not a major regulator of ApoE expression through SphK2/S1P signaling. Interestingly, as previously reported in all-trans retinoic acid-resistant colon cancer cells, SphK2 overexpression resulted in the downregulation of endogenous RXRα (39), but not of LXRα/β (supplemental Fig. S8A–C). Our data suggest that a regulatory mechanism exists between SphK2/S1P signaling and RXRα activity.

### ApoE induced by cotreatment with RXR agonist and SphK2 inhibitor enhances Aβ uptake

**Fig. 7:**
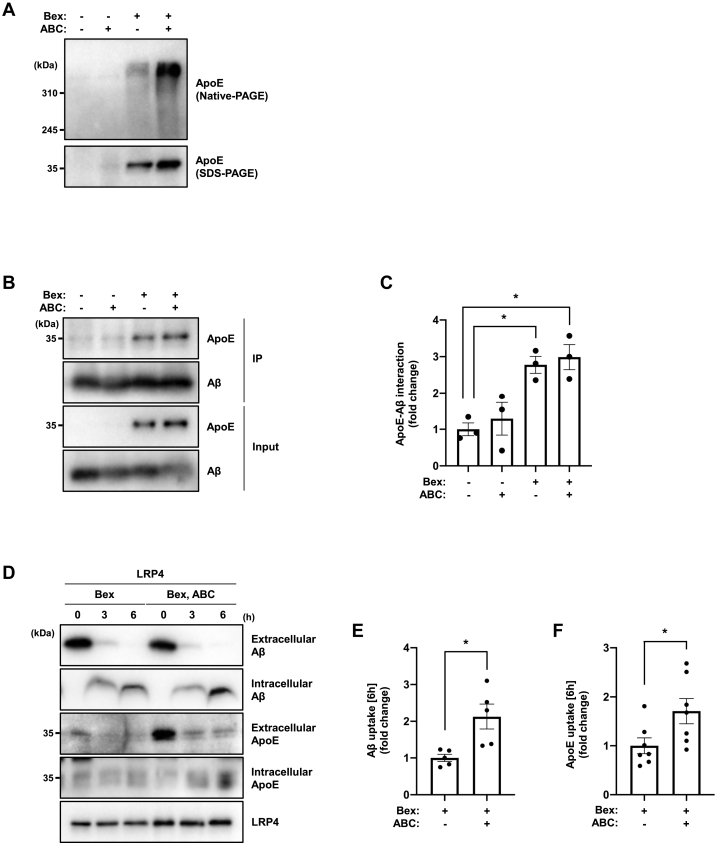
SphK2 inhibitor enhances Aβ uptake in astrocytes. A: Native-PAGE (top) and SDS-PAGE (bottom) analysis for ApoE. U87-control cells were incubated with serum-free medium and treated with 1 μM bexarotene and 3 μM ABC294640 for 48 h. Medium was changed to serum-free 2 h after cell seeding. B: Coimmunoprecipitation analysis using Aβ antibody (4G8). U87-control cells were incubated with serum-free medium and treated with 1 μM bexarotene and 3 μM ABC294640 for 48 h. Then the conditioned medium was incubated with 1 μM synthetic Aβ_1-42_ at 4°C overnight. C: Quantification of secreted ApoE-Aβ interaction shown in Figure 7B. Data are expressed as mean  ±  SEM (*n* = 3, ∗*P* < 0.05: significant difference compared with the vehicle sample by one-way ANOVA with Dunnett’s *post hoc* test). D: Aβ uptake assay in LRP4-expressing U87 cells. U87-control cells were transfected with pFN21AA0816-LRP4-V5-6His and incubated with the medium in which ApoE-Aβ interaction is detected shown in Figure 7B for 6 h. E and F: Quantification of intracellular Aβ (E) and ApoE (F) shown in Figure 7D. Data are expressed as mean ± SEM (*n* = 5–7, ∗*P* < 0.05: significant difference compared with the bexarotene treatment sample by two-tailed Student’s *t*-test). Aβ, amyloid β; ApoE, apolipoprotein E; LRP4, lipoprotein receptor-related protein 4; SphK2, sphingosine kinase 2.

Lipidated ApoE has anti-inflammatory effects and promotes Aβ metabolism (12), and SphK2 inhibitor has anti-inflammatory activity in several diseases (40). These lines of evidence indicate that promoting ApoE induction using SphK2 inhibitors may be a therapeutic strategy for AD. To investigate whether ApoE induced by cotreatment with RXR agonist and SphK2 inhibitor is properly lipidated, we performed native-PAGE analysis. We found that the induced ApoE was similarly lipidated (41, 42), with or without the SphK2 inhibitor (Fig. 7A). Next, to examine the interaction between lipidated ApoE and Aβ, we incubated conditioned medium from reagent-treated U87 cells with synthetic Aβ_1-42_ overnight. Coimmunoprecipitation analysis using Aβ antibody (4G8) revealed that lipidated ApoE could interact with Aβ (Fig. 7B, C). Although the interaction level was similar with or without SphK2 inhibitor, we hypothesized that lipidation status might alter the ApoE and Aβ uptake into the astrocytes. To check this possibility, we focused on LRP4, the major ApoE receptor in astrocytes, to promote Aβ clearance (43). We verified that endocytosis during LRP4 expression was not apparently different from that in the controls using FITC-transferrin (supplemental Fig. S9). The LRP4-V5-6His vector was transiently expressed in U87 cells, and the medium was replaced with one that promotes the ApoE-Aβ complex formation as shown in Figure 7B. Chloroquine was also added to reduce lysosomal degradation. We found that medium from SphK2 inhibitor cotreatment significantly promoted ApoE (1.71-fold) and Aβ (2.13-fold) uptake into cells compared with bexarotene treatment alone (Fig. 7D–F). These results suggest that RXR agonists and SphK2 inhibitors can improve Aβ metabolism in a concerted manner.

## Discussion

Alterations in lipid composition significantly affect the AD pathology, particularly through changes in astrocyte function. Interestingly, the expression of ApoE, which regulates lipid homeostasis, is downregulated in astrocytes in AD (9, 10), which is suggestive of a vicious cycle between abnormal lipid metabolism and aberrant astrocyte function. Furthermore, because ApoE has anti-inflammatory and Aβ-metabolizing effects, increasing ApoE levels may have therapeutic effects on AD. Based on this notion, the induction of ApoE expression by the activation of the nuclear receptors, RXR and LXR, has attracted the attention of researchers. In particular, the therapeutic effect of bexarotene, an RXR agonist, on AD has been well studied, but its effects differ among research groups, suggesting the presence of factors related to AD pathophysiology that modulate the expression or function of ApoE.

Here, we focused on the SphK2/S1P pathway, a lipid signaling pathway, which we previously showed to be activated in AD pathology (17). The signaling lipid S1P is involved in high-density lipoprotein (HDL) function (44) and neuroinflammation through the regulation of gene expression in the nucleus and alteration in the functioning of various immune cells (45). Because ApoE, a risk factor for AD, functionally overlaps with S1P in its involvement in lipid metabolism, neuroinflammatory functions, and related diseases (46), we analyzed the relationship between the two molecules using several astrocyte models. In this study, we analyzed the most common isoform, ApoE3, with the aim of elucidating its functional regulation. Interestingly, we showed that upregulation of SphK2 activity suppressed the induction of ApoE, but not of ABCA1 and ApoJ, by both RXR and LXR agonists at the transcriptional level. We also found that the binding of RXRα to the *APOE* promoter region is suppressed upon SphK2 activation, suggesting the pivotal role of nuclear S1P. Intriguingly, the suppression of SphK2 activity by knockdown or inhibitors upregulated the induction of ApoE and facilitated Aβ uptake in astrocytes. Considering our previous findings that SphK2 activity is upregulated in AD brain and promotes Aβ production in neurons, suppression of SphK2/S1P signaling may be a promising multifunctional therapeutic strategy for AD that can act on both astrocytes and neurons.

Of the two SphK isozymes, SphK2 is predominant in the brain (47). We found a strong correlation between SphK2/S1P activity and ApoE expression; increased SphK2 activity suppressed ApoE expression, and conversely, SphK2 knockdown or inhibitors promoted ApoE expression by RXR or LXR agonist but did not affect ABCA1 and ApoJ expression (Fig. 1, Fig. 2, Fig. 3 and supplemental Fig. S1). ApoJ is one of the main apolipoproteins in the brain and its expression is reported to be upregulated in AD (48). Although the function of ApoJ in AD is not fully understood, the fact that apolipoprotein balance is altered by SphK2/S1P signaling is intriguing and is a subject for future investigation. ApoE regulation by SphK2/S1P signaling was observed in human primary astrocytes and cultured hippocampal slices, suggesting that this mechanism is reproducible in several astrocyte models (Figs. 4 and 5). S1P, a product of SphK2, mainly functions as an extracellular ligand for G protein-coupled receptors and as a transcriptional regulator in the nucleus (49). The sphingosine analog, FTY720, is phosphorylated by SphK2 to produce the S1P analog, FTY720-P. In contrast to S1P, FTY720-P modulates S1P receptors (S1PRs) to reduce their cell surface expression and signaling (50). We found that FTY720 suppressed ApoE induction (supplemental Fig. S4). Considering the effect of SphK2/S1P signaling on *APOE* transcription (Fig. 1, Fig. 2, Fig. 3 and supplemental Fig. S1), the nuclear function of S1P itself is strongly involved in ApoE regulation. Interestingly, we have previously shown that FTY720 increases Aβ_42_ levels in AD model mice (51), and regulation of ApoE expression in astrocytes might be involved and needs to be explored further. However, this study cannot fully deny the non-nuclear function of SphK2/S1P signaling. Indeed, the levels of S1P_3_ receptor are increased and this receptor has been reported to be involved in the induction of inflammation in reactive astrocytes (52). The possibility remains that S1PR signaling, in addition to the nuclear function of SphK2/S1P signaling, cooperatively contributes to altered astrocyte function in AD. Another concern is that in SphK2-expressing hippocampal slices and U87-SphK2 cells, SphK2 was highly localized in the cytoplasm (supplemental Fig. S3 and Fig. 6B). Since S1P is known to shuttle between the cytoplasm and the nucleus, this study leaves open the possibility that S1P acts in the cytoplasm. The nuclear function of SphK2 has been verified in other studies, and further analysis of the function of nuclear S1P will be needed to test our hypothesis in more detail, using the nuclear localization-related mutants described in those articles (53, 54). In addition, our study suggests a correlation between increased S1P and suppressed ApoE expression, but the overall sphingolipid changes were not analyzed. Therefore, this aspect should be investigated in the future.

Interestingly, SphK2/S1P signaling regulated the inflammatory response in astrocytes (supplemental Fig. S5). In particular, activation of the NF-κB pathway by increased SphK2 expression was similar to that observed in regorafenib-resistant hepatocellular carcinoma-derived cells (55). SphK2 expression and nuclear translocation of p65 were increased in these cells. Moreover, *IL6* and *IL1β* expression increased in U87-SphK2 cells (supplemental Fig. S5B, C). Interestingly, activation of the NF-κB pathway by ApoE knockout has also been reported (56). We found that basal *ApoE* levels decreased in U87-SphK2 cells, and ApoE knockdown increased *IL6* expression (supplemental Fig. S5F–H). Considering that S1P and ApoE have proinflammatory and anti-inflammatory effects, respectively, SphK2/S1P signaling may regulate the inflammatory response through the regulation of basal ApoE in astrocytes. However, this study was not able to show a direct relationship between the two, so future verification is needed. In addition, the antitumor effects of SphK2 inhibitors and ApoE have also been reported, so it will be interesting to analyze the relationship between the two in this aspect.

The mechanism underlying the regulation of ApoE expression by SphK2/S1P signaling was not fully elucidated in this study, although there are some clues. Accumulating evidence indicates the importance of S1P as a transcriptional regulator. We confirmed that endogenous SphK2 is predominantly distributed in the nucleus, and its expression level is increased in U87-SphK2 cells (Fig. 6A, B). Although S1P has been reported to repress HDAC activity (34), treatment with an HDAC inhibitor did not affect ApoE production by RXR agonist (supplemental Fig. S7). In addition, because HDAC inhibition has been reported to increase ApoE expression (57), it is unlikely that histone acetylation is involved in this mechanism.

Another hypothesis involves functional regulation of RXR/LXR. The results of ChIP experiments targeting the binding site of RXRα indicated that SphK2/S1P signaling regulates the nuclear RXRα activity (Fig. 6C, D). We also analyzed the expression of nuclear receptors and showed that RXRα expression is decreased in U87-SphK2 cells (supplemental Fig. S8), consistent with previous studies in colon cancer resistant to all-trans retinoic acid, the RXR agonist (39). In contrast, there was no effect of SphK2 overexpression on LXRα/β expression (supplemental Fig. S8). Interestingly, RXRα expression is downregulated in AD model mice (58). Considering that the RXR/LXR heterodimer is activated by RXR agonists alone, SphK2/S1P signaling might act on RXRα directly or indirectly. Since ABCA1, which is also regulated by RXR, is not affected (Fig. 1, Fig. 2, Fig. 3), it is unlikely that the reduced RXRα expression is sufficient to define the expression level of ApoE, and a more complex mechanism is expected.

There are several possible mechanisms underlying the specificity of ApoE. First, the difference in the transcriptional mechanism between *APOE* and *ABCA1* is that *APOE* expression is induced by complex formation with RXR and several transcription factors, not only on the promoter but also on the enhancer region (59, 60), and regulation of the expression of *ABCA1* is simpler than that of *APOE* (61). SphK2/S1P may inhibit the formation of ApoE-specific transcription factor complexes. The second mechanism is a conformational change in RXRα. Recently, modulators that alter the conformation of nuclear receptors, thereby modulating their function, have attracted considerable attention (62). Interestingly, the effects of S1P on nuclear receptors, such as peroxisome proliferator-activated receptor γ (63), are gradually being revealed, and it will be necessary to verify whether S1P directly affects the conformation and activity of RXRα.

A role for SphK2/S1P signaling in AD has been proposed. We have shown an increase in SphK2 activity in the frontal cortex of AD brains (17). Notably, Lei *et al.* reported that SphK2 KO J20 mice have decreased Aβ aggregation (19). Another group, using mice deficient in SGPL1, an irreversible S1P-degrading enzyme, reported that S1P accumulation induces neurodegeneration, tau pathology, and astrogliosis (18, 64). However, some groups have shown a reduction in SphK2 activity or S1P levels in AD (65, 66). It is important to discuss these conflicting findings, including the localization of SphK2. SphK2 has both nuclear localization and export signals for shuttling between the cytoplasm and nucleus in response to the cellular environment (53). Cytoplasmic SphK2-derived S1P has cytoprotective effects, such as promoting cell survival; however, under stress conditions, such as high cell density or serum starvation, it accumulates in the nucleus (53, 67). Indeed, the nuclear accumulation of SphK2 has been reported in AD brains (36), suggesting that the harmful effect of nuclear S1P plays an important role in AD pathogenesis. Our results may facilitate the development of novel AD treatment strategies. However, SphK2 KO J20 mice showed hippocampal volume loss, myelin loss, and cognitive impairment (19); therefore, the possibility of adverse effects should be considered.

Lipidated ApoE exists extracellularly as HDL-like particles. S1P is incorporated into HDL-like particles in an ABCA1-dependent manner and coexists with ApoE (44). These particles regulate various physiological functions, such as Aβ uptake via ApoE receptors and cell migration via S1PRs (68, 69). ApoE secreted upon cotreatment with RXR agonist and SphK2 inhibitor was lipidated as in the case of treatment with RXR agonist alone (Fig. 7A). Although the efficiency of binding between ApoE and Aβ was not significantly different (Fig. 7B, C), we hypothesized that ApoE induced by cotreatment with RXR agonist and SphK2 inhibitor would form lipid particles lacking S1P and would alter its function.

In this study, we focused on the ApoE receptor, LRP4. LRP4 is the most abundant among the LDLR family members known as ApoE receptors in astrocytes (70). Furthermore, LRP4 is downregulated in the hippocampus, cortex, and entorhinal cortex of AD patients, and amyloid pathology is exacerbated in 5×FAD mice lacking LRP4, suggesting an important role for LRP4 in Aβ metabolism (43). In the present study, we found that cotreatment with RXR agonist and SphK2 inhibitor significantly enhanced uptake of Aβ as well as ApoE in LRP4-expressing astrocytes, suggesting a protective effect via enhancement of Aβ metabolism (Fig. 7D–F). We also demonstrate that the combination of an RXR agonist and a SphK2 inhibitor is effective for the treatment of AD. In this study, chloroquine was used to evaluate uptake, which does not fully reflect physiological conditions because Aβ is inherently degraded. While this analysis has revealed an enhancement effect of Aβ uptake, it will be necessary to clarify the relationship between LRP4 and Aβ under pathological conditions, such as in AD model mice in the future. Bexarotene has shown therapeutic effects in AD model mice (11), and clinical trials have shown reduction in cortical amyloid levels, although side effects, particularly triglyceride accumulation, have been observed (71). However, the effects of bexarotene have been variable in most follow-up studies (13, 14, 15, 16), suggesting that certain factors cause these variations. Our results suggest that enhanced SphK2/S1P signaling may be a contributing factor. Moreover, SphK2 inhibitors may compensate for the effects of bexarotene, requiring lower doses and fewer adverse effects.

ApoE has three isoforms—ApoE2, ApoE3, and ApoE4—with ApoE3 being the most common. Although the products of these gene variants differ by only one or two amino acid residues, these small differences have profound effects on binding to Aβ, ApoE receptors, and lipids (46). In particular, ApoE4 is known to be the strongest genetic risk factor for sporadic AD, and the number of ε4 genes affects the risk and accelerates the onset of AD (72). Interestingly, bexarotene has been shown to be less effective against ApoE4 carriers than noncarriers (71). In addition, it has been reported that the promotion of ApoE4 lipidation suppresses ApoE4-driven pathology (42, 73). Similarly, SphK2 inhibitors increase lipidated ApoE levels and may also promote Aβ metabolism under ApoE4 conditions. However, it has been reported that ApoE4 promotes AD pathology through toxic gain of function (74). As our study focused on ApoE3, we must investigate the effect of ApoE4 in the treatment of AD.

In conclusion, in this study, we demonstrate the importance of nuclear S1P in astrocyte function. It may work in concert with known therapeutic targets, RXR and LXR agonists, to increase ApoE and improve astrocyte function, potentially expanding treatment options. Furthermore, considering our previous studies (17), SphK2/S1P signaling is a potential target for multitargeted therapy, that is, Aβ production in neurons and Aβ metabolism via the regulation of ApoE expression in astrocytes. Although the relationship between nuclear lipid function and AD pathogenesis remains unclear, this study may provide a clue to this question, and a comprehensive search for nuclear S1P-binding proteins in the future will be useful in understanding the functional changes of astrocytes in AD.

## Data Availability

All data are contained within the article and the supporting information. All data reported in this manuscript will be shared by the lead contact upon request. Raw data of immunoblotting, RT-PCR and ChIP in main figures are deposited on https://doi.org/10.17632/rb89bxdhwj.2.

## Supplemental data

This article contains supplemental data.

## Conflict of interest

The authors declare that they have no conflicts of interest with the contents of this article.
