# Supplementary material for: Nuclear SphK2/S1P signaling is a key regulator of ApoE production and Aβ uptake in astrocytes
Source: J Lipid Res. 2024 Jan 26;65(3):100510. doi: 10.1016/j.jlr.2024.100510 (PMC10907773; doi:10.1016/j.jlr.2024.100510)
Supplement: Supplemental Figure [file mmc1.pdf]

Fig. S1

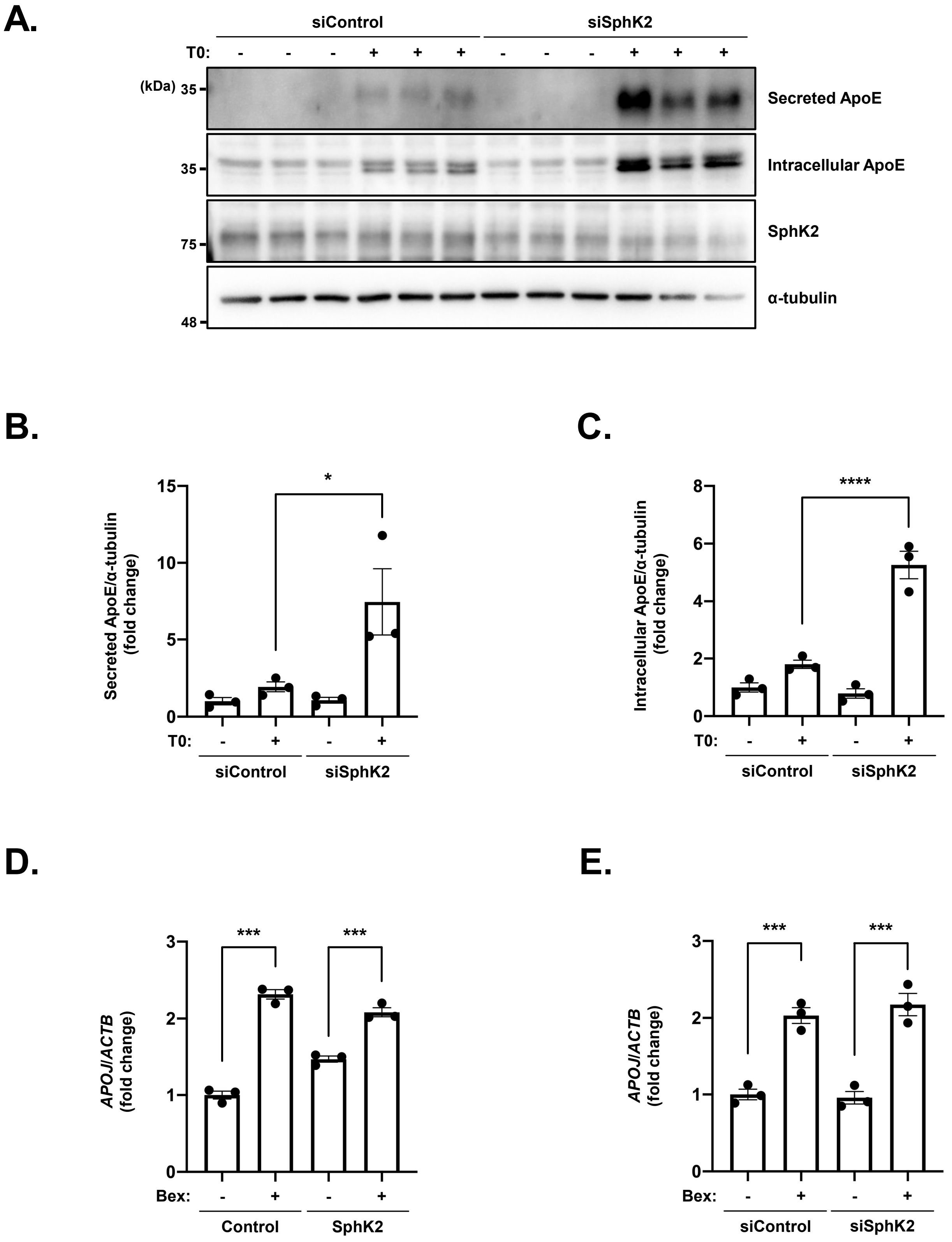
A.
siControl
siSphK2
T0:
(kDa)
35
35
75
48
Secreted ApoE
Intracellular ApoE
SphK2
α-tubulin
B.
Secreted ApoE/α-tubulin (fold change)
T0:
siControl
siSphK2
C.
Intracellular ApoE/α-tubulin (fold change)
T0:
siControl
siSphK2
D.
APOJ/ACTB (fold change)
Bex:
Control
SphK2
E.
APOJ/ACTB (fold change)
Bex:
siControl
siSphK2

Supplemental Fig. S1 SphK2 knockdown increases ApoE induction by LXR agonist.

A: Immunoblotting analysis for ApoE and SphK2 in control and SphK2 siRNA treated cells. U87-control cells were transfected with siRNA and after 24 h, treated with 1 μM T0901317 for 48 h. Medium was changed to serum-free 6 h after transfection.

B and C: Quantification of secreted (B) and intracellular (C) ApoE in control and SphK2 siRNA treated cells shown in Fig. S1A. The relative protein levels were normalized to those of α-tubulin. Data are expressed as mean ± SEM ($n = 3$, *$P < 0.05$, ****$P < 0.0001$: significant difference compared with the control sample treated with T0901317 by one-way ANOVA with Dunnett's *post hoc* test).

D: Quantification of *APOJ* mRNA in U87-control and U87-SphK2 cells by RT-qPCR analysis. Cells were incubated with serum-free medium and treated with 1 μM bexarotene for 48 h. Medium was changed to serum-free 2 h after cell seeding. The relative mRNA levels were normalized to those of *ACTB*. Data are expressed as mean ± SEM ($n = 3$, ***$P < 0.001$: significant difference compared with the vehicle sample in U87-control or U87-SphK2 cells by one-way ANOVA with Bonferroni's *post hoc* test).

E: Quantification of *APOJ* mRNA in control and SphK2 siRNA treated cells by RT-qPCR analysis. U87-control cells were transfected with siRNA and after 24 h, treated with 1 μM bexarotene for 48 h. Medium was changed to serum-free 6 h after transfection. The relative mRNA levels were normalized to those of *ACTB*. Data are expressed as mean ± SEM ($n = 3$, ***$P < 0.001$: significant difference compared with the vehicle sample by one-way ANOVA with Bonferroni's *post hoc* test).

ACTB, actin beta; ApoE, apolipoprotein E; ApoJ, apolipoprotein J; RT-qPCR, reverse transcription-quantitative PCR; SphK2, sphingosine kinase 2.

Fig. S2

A.

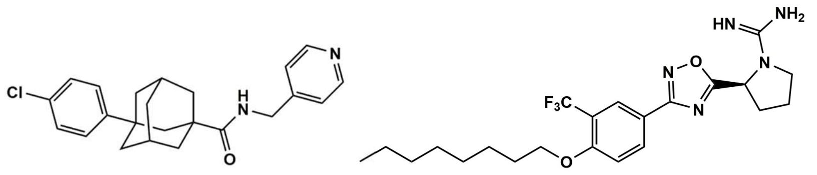

ABC294640 SLM6031434

B.

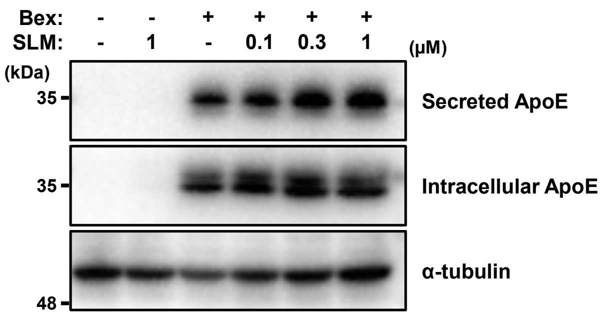

C.

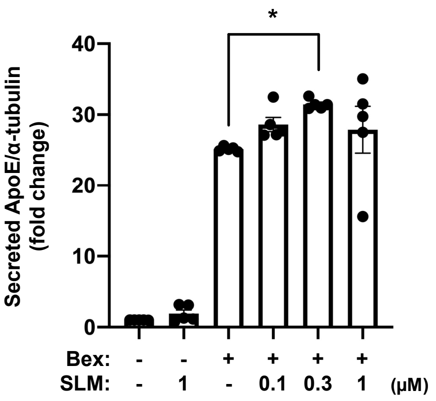

Supplemental Fig. S2 SphK2 inhibitor increases ApoE induction by RXR agonist.

A: Chemical structure of ABC294640 (left) and SLM6031434 (right). Each structure was drawn by ChemDraw Professional 16.0.

B: Immunoblotting analysis for ApoE under the SphK2 inhibition condition. U87-control cells were incubated with serum-free medium and treated with 1 μM bexarotene and the indicated concentration of SLM6031434 (SLM) for 48 h. Medium was changed to serum-free 2 h after cell seeding.

C: Quantification of secreted ApoE shown in Fig. S2B. The relative protein levels were normalized to those of α-tubulin. Data are expressed as mean ± SEM ($n = 5$, $*P < 0.05$: significant difference compared with the bexarotene sample by one-way ANOVA with Dunnett's *post hoc* test).

ApoE, apolipoprotein E; RXR, retinoid X receptor; SphK2, sphingosine kinase 2.

Fig. S3

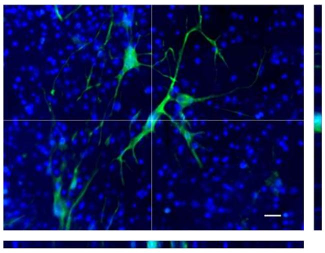

Supplemental Fig. S3 Expressed SphK2 is localized in nucleus.

Immunostaining of hippocampal slice. V5-6His tagged SphK2 (green) and nuclear marker DAPI (blue) were detected. Slices were incubated with serum-free medium consisting of MEM, HBSS and B-27-supplemented Neurobasal-A and analyzed at DIV11. Representative z-stack images were captured at 0.3 μm intervals along the z-axis using a 60× objective oil lens. Scale bars represent 20 μm.

DAPI, 4',6-diamidino-2-phenylindole; DIV, days in vitro; HBSS, Hanks' balanced salt solution; MEM, minimum essential medium; SphK2, sphingosine kinase 2.

Fig. S4

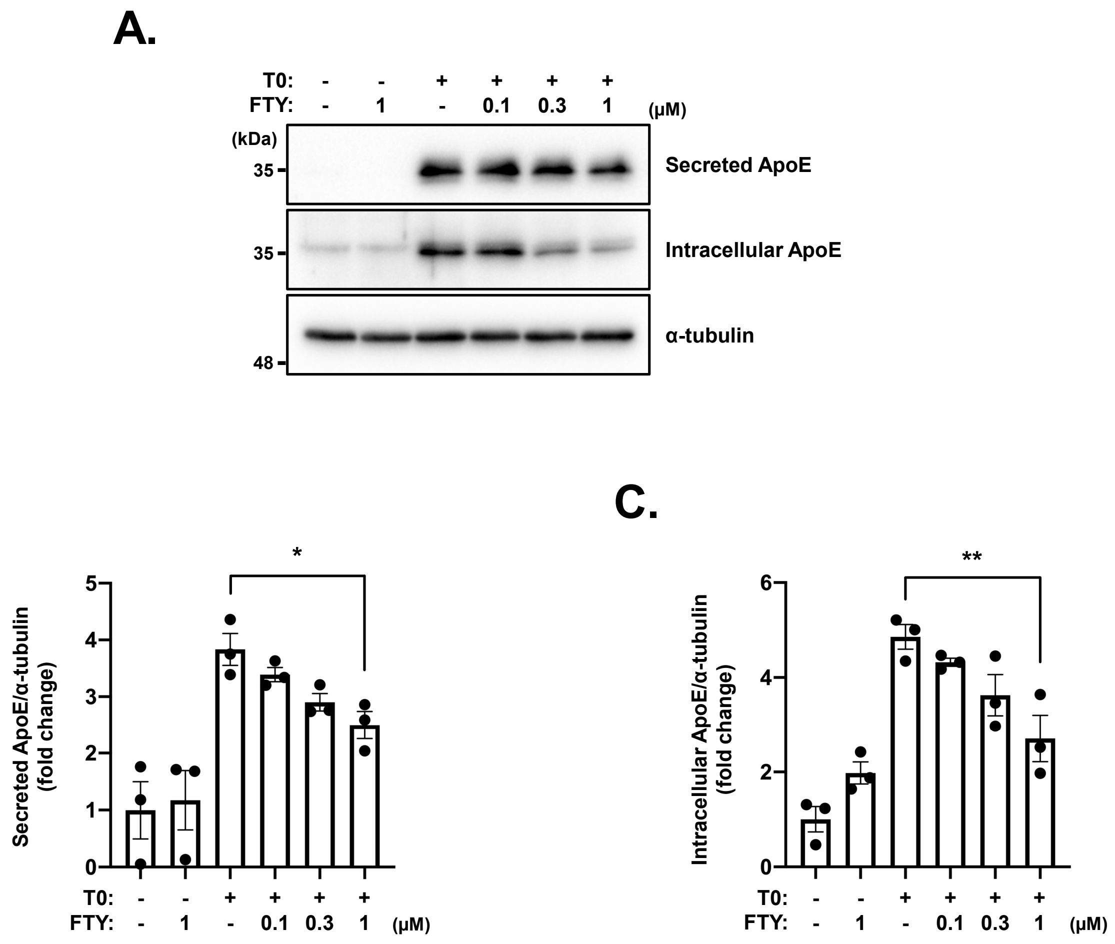

Supplemental Fig. S4 FTY720 suppresses ApoE induction by LXR agonist.

A: Immunoblotting analysis for ApoE. U87-control cells were incubated with serum-free medium and treated with 1 μM T0901317 and the indicated concentration of FTY720 (FTY) for 48 h. Medium was changed to serum-free 2 h after cell seeding.

B and C: Quantification of secreted (B) and intracellular (C) ApoE shown in Fig. S4A. The relative protein levels were normalized to those of α-tubulin. Data are expressed as mean ± SEM ($n = 3$, *P < 0.05, **P < 0.01: significant difference compared with the T0901317 treatment sample by one-way ANOVA with Dunnett's *post hoc* test).

ApoE, apolipoprotein E.

Fig. S5

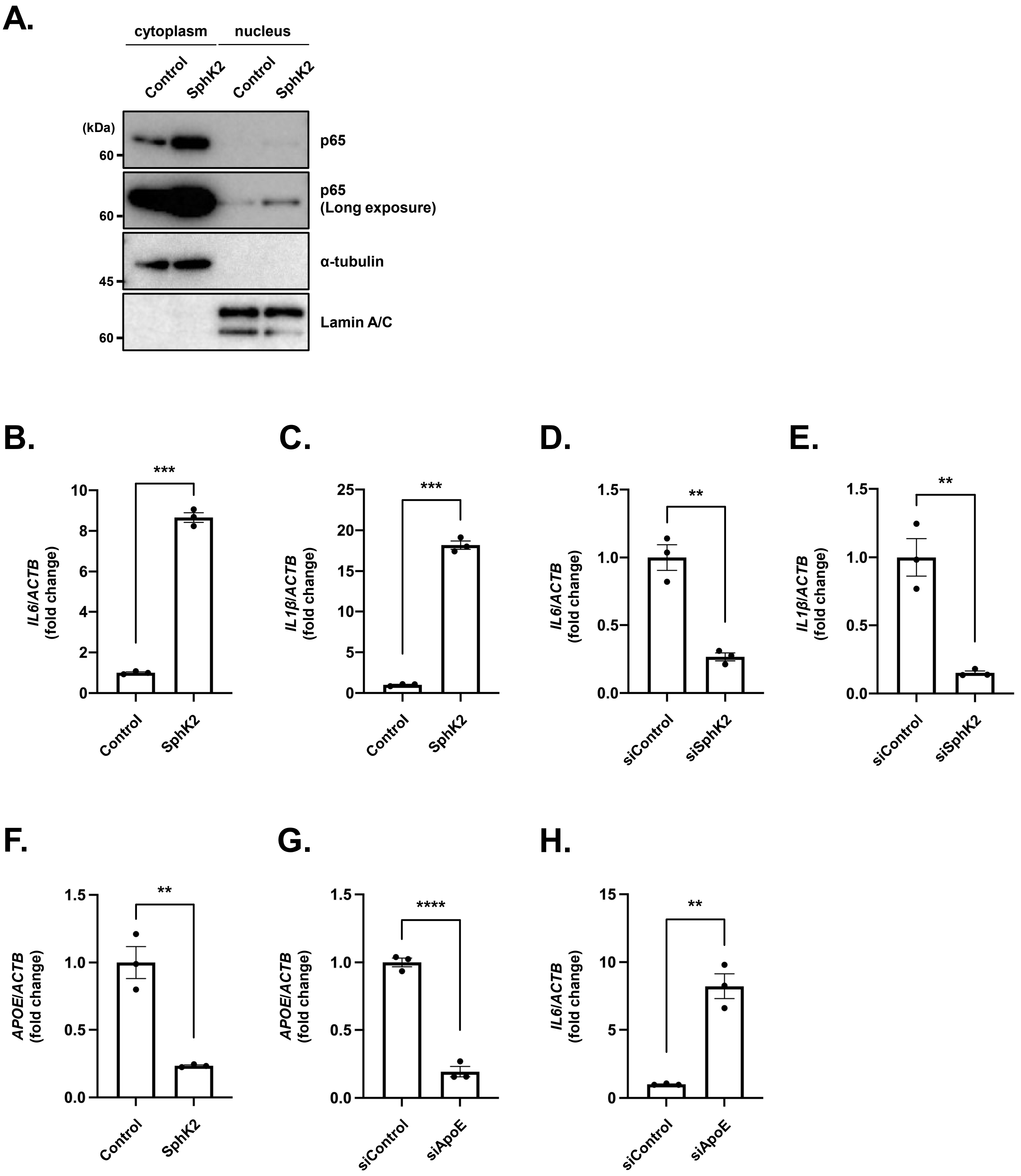
A.
cytoplasm
nucleus
Control
SphK2
Control
SphK2
(kDa)
60
60
45
60
p65
p65
(Long exposure)
α-tubulin
Lamin A/C
B.
IL6/ACTB
(fold change)
Control
SphK2
***
C.
IL1β/ACTB
(fold change)
Control
SphK2
***
D.
IL6/ACTB
(fold change)
siControl
siSphK2
**
E.
IL1β/ACTB
(fold change)
siControl
siSphK2
**
F.
APOE/ACTB
(fold change)
Control
SphK2
**
G.
APOE/ACTB
(fold change)
siControl
siApoE
****
H.
IL6/ACTB
(fold change)
siControl
siApoE
**

Supplemental Fig. S5 SphK2/S1P signaling regulates astrocyte reactivity.

A: Nuclear fractionation analysis for NF-κB p65. U87-control and U87-SphK2 cells were incubated with serum-free medium. α-tubulin and Lamin A/C were used as cytoplasm and nuclear marker protein, respectively.

B and C: Quantification of *IL6* (B) and *IL1β* (C) mRNA in U87-control and U87-SphK2 cells by RT-qPCR analysis. Cells were incubated with serum-free medium. The relative mRNA levels were normalized to those of *ACTB*. Data are expressed as mean ± SEM ($n = 3$, ***$P < 0.001$: significant difference compared with the U87-control sample by two-tailed Student's *t*-test).

D and E: Quantification of *IL6* (D) and *IL1β* (E) mRNA in control and SphK2 siRNA treated cells by RT-qPCR analysis. U87-control cells were transfected with siRNA and medium was changed to serum-free. The relative mRNA levels were normalized to those of *ACTB*. Data are expressed as mean ± SEM ($n = 3$, **$P < 0.01$: significant difference compared with the control sample by two-tailed Student's *t*-test).

F: Quantification of basal *APOE* mRNA in U87-control and U87-SphK2 cells using Figure 1H data. The relative mRNA levels were normalized to those of *ACTB*. Data are expressed as mean ± SEM ($n = 3$, **$P < 0.01$: significant difference compared with the U87-control sample by two-tailed Student's *t*-test).

G and H: Quantification of *APOE* (G) and *IL6* (H) mRNA in control and ApoE siRNA treated cells by RT-qPCR analysis. U87-control cells were transfected with siRNA and medium was changed to serum-free. The relative mRNA levels were normalized to those of *ACTB*. Data are expressed as mean ± SEM ($n = 3$, **$P < 0.01$, ****$P < 0.0001$: significant difference compared with the siControl sample by two-tailed Student's *t*-test).

ACTB, actin beta; ApoE, apolipoprotein E; IL1β, interleukin 1β; IL6, interleukin 6; RT-qPCR, reverse transcription-quantitative PCR; S1P, sphingosine-1-phosphate; SphK2, sphingosine kinase 2.

Fig. S6

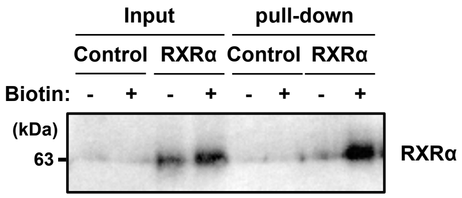

Supplemental Fig. S6 Confirmation of RXRα pull-down efficiency.

Immunoblotting analysis for RXRα. U87-control cells were transfected with pCAGGS-BLRP-IRES-BirA (control) or pCAGGS-BLRP-hRXRα-IRES-BirA (RXRα) and after 24 h, treated with 50 μg/ml D-biotin along with medium change to serum-free condition for 24 h. Then, the lysates were pull-downed using NeutrAvidin Agarose.

BLRP, biotin ligase recognition peptide; RXR, retinoid X receptor.

Fig. S7

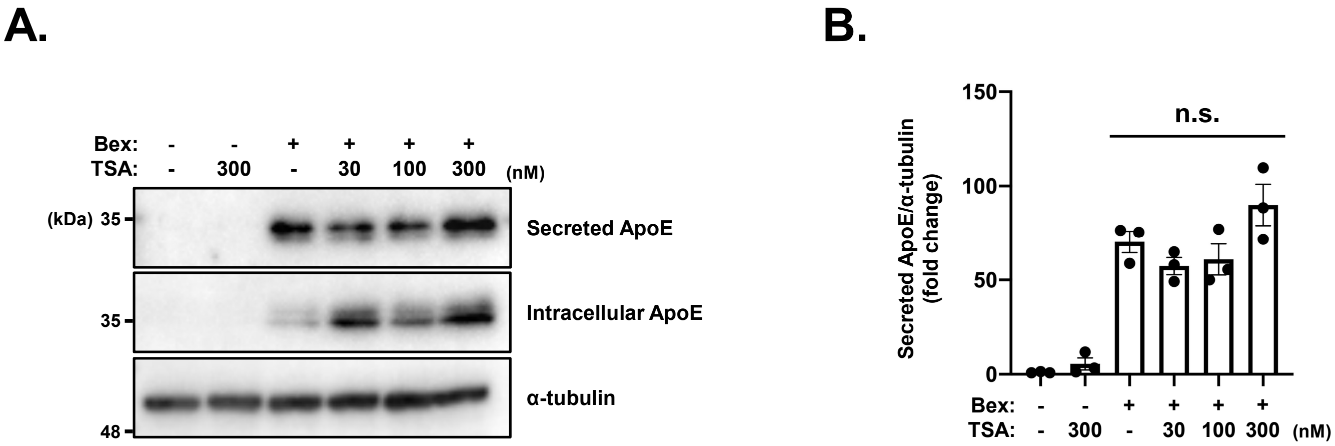

Supplemental Fig. S7 Histone modification is not involved in ApoE regulation by SphK2/S1P signaling.

A: Immunoblotting analysis for ApoE under the inhibition of HDAC activity. U87-control cells were incubated with serum-free medium and treated with 1 μM bexarotene for 48 h. Cells were pretreated with trichostatin A (TSA) for 24 h before stimulation with bexarotene. The medium was changed to serum-free along with TSA treatment.

B: Quantification of secreted ApoE shown in Fig. S7A. The relative protein levels were normalized to those of α-tubulin. Data are expressed as mean ± SEM (n = 3, n.s.= not significant: significant difference compared with the bexarotene treatment sample by one-way ANOVA with Dunnett's *post hoc* test).

ApoE, apolipoprotein E; HDAC, histone deacetylase; S1P, sphingosine-1-phosphate; SphK2, sphingosine kinase 2.

Fig. S8

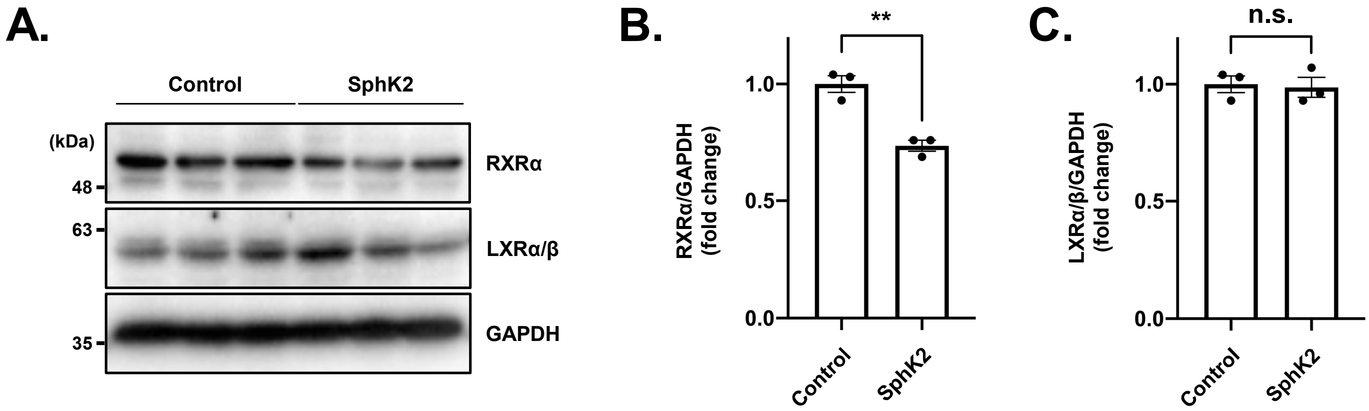

Supplemental Fig. S8 SphK2/S1P signaling affects RXRα expression.

A: Immunoblotting analysis for RXRα and LXRα/β in U87-control and U87-SphK2 cells.

B and C: Quantification of RXRα (B) and LXRα/β (C) shown in Fig. S8A. The relative protein levels were normalized to those of GAPDH. Data are expressed as mean ± SEM ($n = 3$, $^{**}P < 0.01$, n.s.= not significant: significant difference compared with the U87-control by two-tailed Student's *t*-test).

RXR, retinoid X receptor; S1P, sphingosine-1-phosphate; SphK2, sphingosine kinase 2.

Fig. S9

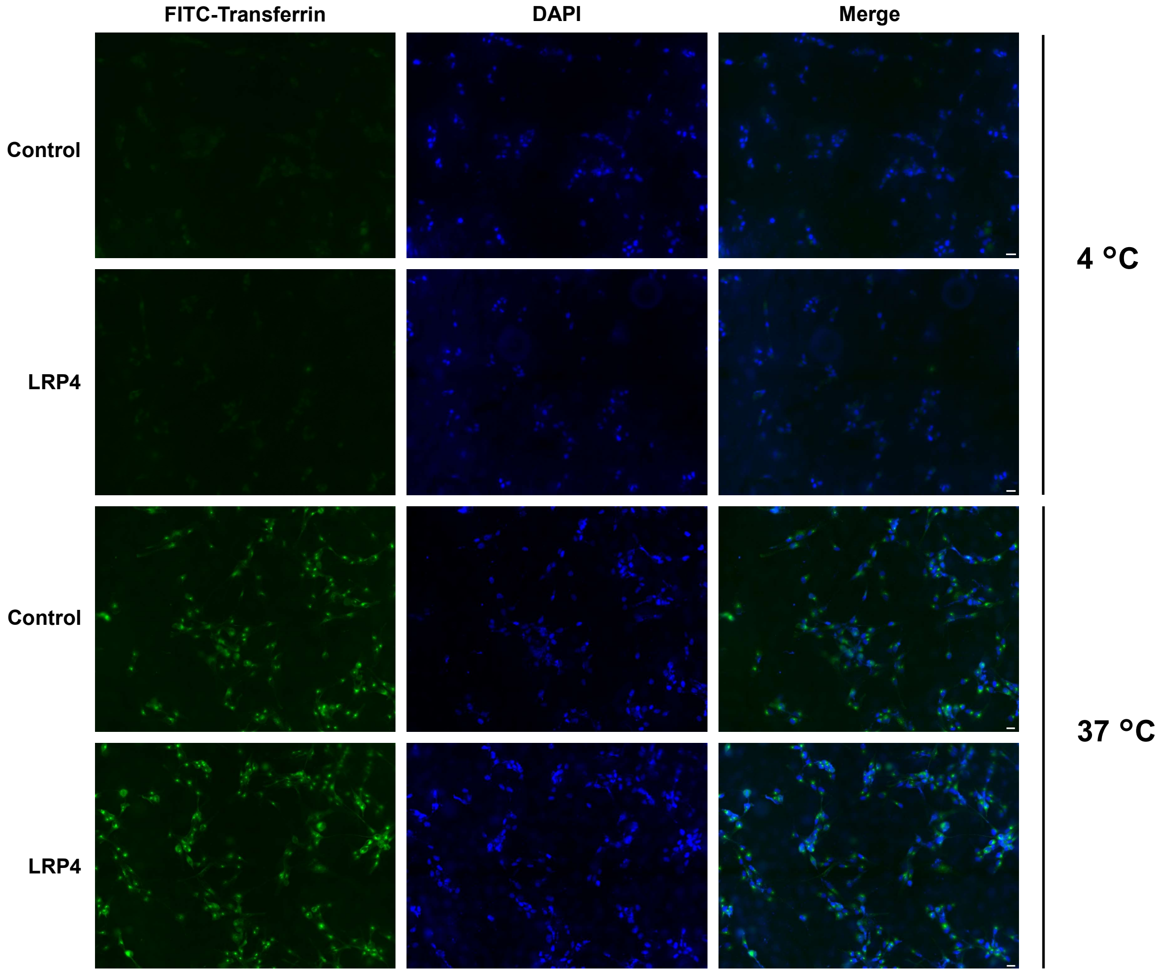

Supplemental Fig. S9　LRP4 does not affect transferrin uptake.

Transferrin uptake analysis in control and LRP4-expressing U87 cells. Cells were transfected with pFN21AA0816-LRP4-V5-6His. Following day, medium was changed to serum-free and cells were incubated for 1 h. Medium was replaced with medium containing FITC-Transferrin and incubated at 37°C for 30 min. The cells were preincubated at 4°C for 10 min, and FITC-transferrin was added in the same manner and incubated at 4°C for 30 min as a control. Scale bars represent 20 μm.

LRP4, lipoprotein receptor-related protein 4.
